# Degradation of Sulfadiazine by Biogenic Manganese Oxides Coupled with Syringaldehyde: Performance, Mechanism, Toxicity, and Environmental Applicability

**DOI:** 10.3390/molecules31142484

**Published:** 2026-07-16

**Authors:** Yifei Leng, Jiyi Wang, Fengyi Chang, Zhu Li, Buyun Wu, Bangding Han, Yu Huang, Wen Xiong

**Affiliations:** 1 Key Laboratory of Intelligent Health Perception and Ecological Restoration of Rivers and Lakes, Ministry of Education, Hubei University of Technology, Wuhan 430068, China; chang602118@163.com (F.C.); iamlizhu@hbut.edu.cn (Z.L.); huangyu@hbut.edu.cn (Y.H.); 2 Cooperative Innovation Center of Industrial Fermentation, Ministry of Education & Hubei Province, Hubei University of Technology, Wuhan 430068, China; 3 School of Civil Engineering, Architecture and Environment, Hubei University of Technology, Wuhan 430068, China; fmvphno3@gmail.com (J.W.); wby12316@outlook.com (B.W.); hbuthanbangding@outlook.com (B.H.); 4 Key Laboratory of Groundwater Quality and Health, China University of Geosciences, Ministry of Education, Wuhan 430078, China

**Keywords:** biological manganese oxide, redox mediator, sulfadiazine, degradation, mechanism

## Abstract

The ecological risks brought by sulfadiazine (SDZ) residues in the environment have put forward requirements for efficient antibiotic treatment technologies. In this study, biogenic manganese oxides (BMOs) were synthesized using the bacterium *Stenotrophomonas maltophilia* DT1, and a BMO/syringaldehyde (SYR) system was constructed for SDZ degradation to investigate its performance, mechanism, and potential application. Results showed that the DT1-synthesized BMO contained Mn (II/III/IV) and defect-related oxygen species, which enabled the BMO to participate in SYR activation and SDZ transformation. A total of 99.67% of 10 mg/L SDZ was removed within 3 h under optimized conditions. Humic acid and most environmental ions had no significant interference with the system, except for slight inhibition by Fe^3+^ and Mn^2+^. Three degradation pathways of SDZ were elucidated through the identification of five transformation products and density functional theory calculations. ECOSAR toxicity prediction and growth inhibition of *Escherichia coli* revealed that the degradation products exhibited significantly reduced toxicity. Furthermore, the BMO exhibited 4.41–17.47 times higher SYR-mediated SDZ transformation efficiency than chemically synthesized manganese oxide (CMO) and showed good performance in reuse tests and real water matrices. This study provides an efficient and eco-friendly green technology for the remediation of SDZ pollution in aquatic environments and provides potential support for the removal of refractory pollutants mediated by BMO.

## 1. Introduction

Sulfadiazine (SDZ), a typical sulfonamide antibiotic, is widely used in human medicine, veterinary treatment, and agricultural disease management owing to its broad antibacterial spectrum, low cost, and stable chemical properties [1]. However, over 50% of the antibiotic is excreted in its parent or metabolite form due to its incomplete metabolism in organisms, subsequently entering soil and aquatic environments via agricultural runoff, livestock manure application, and domestic sewage discharge [2]. Environmental monitoring studies have detected SDZ at μg/L levels in surface water, groundwater, and drinking water sources and up to several mg/kg in manure-amended soils [1]. The long-term environmental persistence of SDZ poses substantial risks, including the induction of antibiotic resistance gene formation and dissemination in microorganisms, disruption of aquatic and soil microbial community equilibrium [3], and exhibiting acute/chronic toxicity to non-target organisms, such as inhibiting algae growth, causing daphnia developmental abnormalities, and accumulating in fish tissues [4]. Therefore, the development of efficient and eco-friendly technologies for SDZ removal from contaminated environments is urgently required.

Many studies have reported SDZ removal technologies, including membrane filtration, adsorption, radiolysis, chlorination, Fenton processes, photocatalysis, and enzyme-mediated oxidation. However, these methods for pollutant removal suffer from limitations such as high cost and the potential for secondary pollution [5]. Recently, biogenic manganese oxides (BMOs) biosynthesized by manganese-oxidizing bacteria have attracted considerable attention due to their strong oxidation potential and green synthetic routes [6]. These microorganisms typically harbor multicopper oxidase genes, which enable the oxidation of Mn(II) to Mn(IV) under appropriate environmental conditions, thereby forming BMO [7]. In addition, compared with chemically synthesized manganese oxides, the biological oxidation of Mn(II) is not only several orders of magnitude faster than abiotic Mn(II) oxidation but also exhibits reaction efficiency toward organic contaminants [8]. BMO primarily consists of amorphous or poorly crystalline nanoparticles, characterized by a large specific surface area, abundant surface defects, and high-density reactive sites, which confer strong adsorption capacity and redox reactivity [9]. Previous studies have demonstrated that BMO can effectively remove oxytetracycline [10], phenol [11], and steroid hormones [11,12] via adsorption and degrade antibiotic drugs such as ciprofloxacin [13], sulfamethoxazole [14], and tetracycline [15] via direct oxidation. For instance, Liang et al. utilized the strain *Achromobacter* sp. JL9 to achieve sulfamethoxazole removal via microbially mediated manganese oxidation within 84 h [14]. Nevertheless, BMO used alone often requires extended reaction durations or high dosages to achieve satisfactory removal efficiency [16].

Enzymatic oxidation has also been widely investigated for the removal of antibiotics and other organic pollutants under mild conditions. Among oxidative enzymes, laccases can catalyze the one-electron oxidation of a wide range of substrates, while molecular oxygen acts as the terminal electron acceptor and is reduced to water during the catalytic cycle [17]. However, the oxidative capacity of enzymes alone is sometimes insufficient for the transformation of pollutants with relatively high redox potentials or complex molecular structures. Therefore, redox mediators can be introduced into enzymatic systems to improve the apparent redox capacity and electron-transfer efficiency of the reaction system [18]. Specific small molecules, termed redox mediators, can be oxidized by enzymes (e.g., laccase, peroxidase) to generate free radicals, thereby increasing the redox potential of the system and improving the degradation efficiency of organic contaminants [19]. Compared with pure enzyme systems, this mediated approach not only enhances reaction efficiency but also expands the substrate scope. Previous work reported that tetracycline degradation increased by approximately 40% using horseradish peroxidase (HRP) coupled with the redox mediator 2,2′-azinobis (3-ethylbenzothiazoline-6-sulfonic acid), relative to the pure enzyme system [20]. The degradation rate of norfloxacin by HRP increased from 0% to nearly 80% following the addition of p-coumaric acid [21]. Moreover, BMO has also been combined with other technologies to enhance pollutant degradation. For example, BMO can mediate ofloxacin degradation via catalyzing heterogeneous photocatalytic Fenton processes [22] and activate peroxymonosulfate to degrade 2,4-dimethylaniline [23]. Although previous studies have confirmed the potential of BMO or enzyme–mediator systems for antibiotic removal, the coupling of BMO with a redox mediator to enhance SDZ transformation has not been reported, and the underlying transformation mechanism remains unclear. Therefore, the novelty of this study lies in constructing a BMO/SYR system for SDZ transformation and clarifying the role of BMO-mediated SYR activation in the formation of SYR-derived reactive intermediates. Unlike previous studies focusing on BMO alone or enzyme–mediator systems, this work integrates biogenic manganese oxides with a phenolic redox mediator and systematically elucidates SDZ transformation pathways and toxicity changes. Moreover, the environmental applicability of the BMO/SYR system was preliminarily evaluated in different water matrices, which may provide a basis for exploring its possible engineering application in the future.

Hence, the objectives of this study were: (1) to synthesize and characterize nanostructured BMO from a potential manganese-oxidizing bacterium *Stenotrophomonas maltophilia* DT1; (2) to investigate the effects of key parameters (mediator type, pH, mediator concentration, BMO dosage, temperature, and SDZ concentration) and environmental factors (humic acid and coexisting ions) on SDZ removal efficiency; (3) to identify SDZ transformation products, elucidate degradation pathways, and perform theoretical calculations and a conceptual mechanism; (4) to evaluate the toxicity of byproducts using ECOSAR software (version 2.2) and *Escherichia coli* growth inhibition assays; (5) to assess the application prospects of the BMO/SYR system. The findings of this study are expected to facilitate the development of a novel, efficient, and eco-friendly technology for SDZ remediation, while providing theoretical insights into mediator-enhanced BMO systems for the degradation of refractory organic pollutants.

## 2. Results and Discussion

### 2.1. Identification and Characterization of BMO

**Figure 1:**
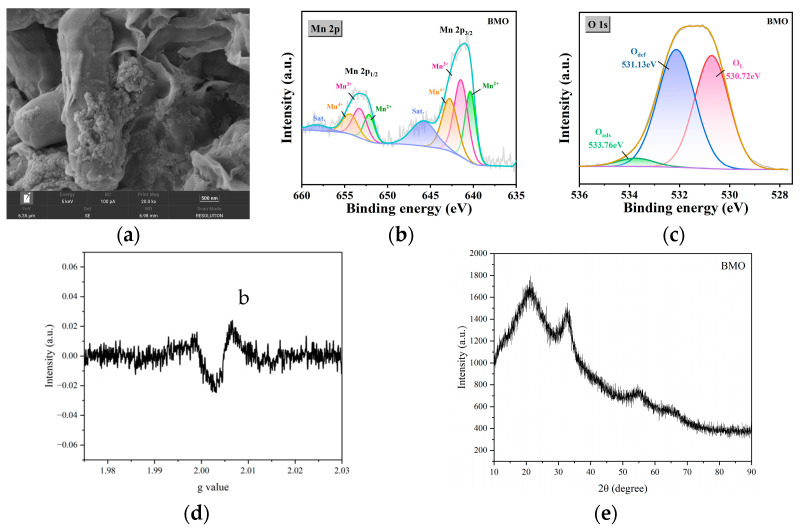
SEM images (**a**), XPS spectra (Mn2p (**b**) and O1s (**c**)), EPR (**d**) and XRD (**e**) of BMO.

Previous genomic analysis found that *S. maltophilia* DT1 harbors a multicopper oxidase gene [3], indicating its potential for BMO biosynthesis. During 7 days of cultivation in PCYM, DT1 cells gradually changed color from yellow to brown; this phenomenon was consistent with BMO formation (Figure S1a,b). These brown cells were capable of converting the LBB solution from colorless to blue, which meant the successful formation of BMO (Figure S1c). The SEM images (Figure 1a) show that the surface of DT1 cells was densely coated with small particles and loose, porous structures, which were consistent with observations from a previous study [24]. EDS mapping (Figures S2 and S3) confirmed that these deposits were rich in manganese and oxygen elements, indicating a high content of manganese oxides.

The high-resolution XPS analysis was further performed to qualitatively identify the oxidation states and surface oxygen species of BMO. Gaussian–Lorentzian fitting of the Mn2p and O1s spectra qualitatively verified the coexistence of multiple manganese oxidation states and diverse oxygen species on the BMO surface (Figure 1b,c). The Mn2p spectrum exhibited characteristic peaks assigned to Mn(II), Mn(III), and Mn(IV) species at 640.4 eV, 641.5 eV, and 642.7 eV, respectively, demonstrating the presence of mixed-valence manganese on BMO [9,25]. Meanwhile, the O1s spectrum was deconvoluted into lattice oxygen (O_L_, 530.7 eV), defect-related oxygen (O_def_, 532.1 eV), and adsorbed oxygen (O_ads_, 533.8 eV). The coexistence of mixed-valence Mn species and abundant defect/adsorbed oxygen species sufficiently demonstrates the oxidizing property of the BMO material [25,26]. The weak EPR signal (Figure 1d) provides qualitative auxiliary evidence for the existence of defect-derived paramagnetic centers in BMO. The XRD pattern of BMO (Figure 1e) indicated a poorly crystalline structure, which was consistent with previous reports [16]. In conclusion, these results indicate that BMO contains defect-related centers and a poor crystalline structure. Meanwhile, the relatively high abundance of Mn(III) and Mn(IV) might enhance the electron acceptance capacity and redox flexibility of BMO [16]. These structural features of BMO lay the foundation for its excellent oxidative activity.

### 2.2. Influencing Factors of SDZ Removal by BMO/Redox Mediator System

**Figure 2:**
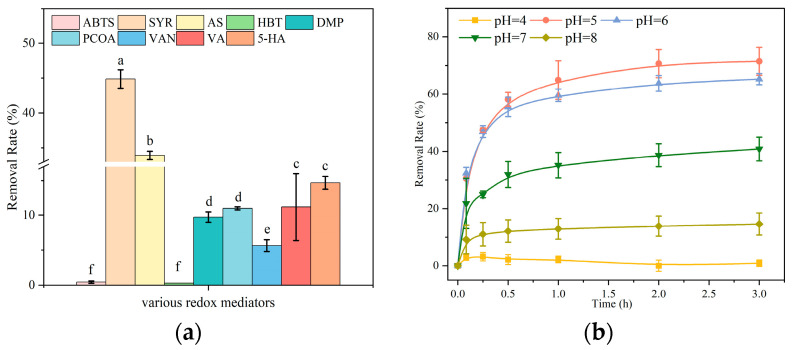

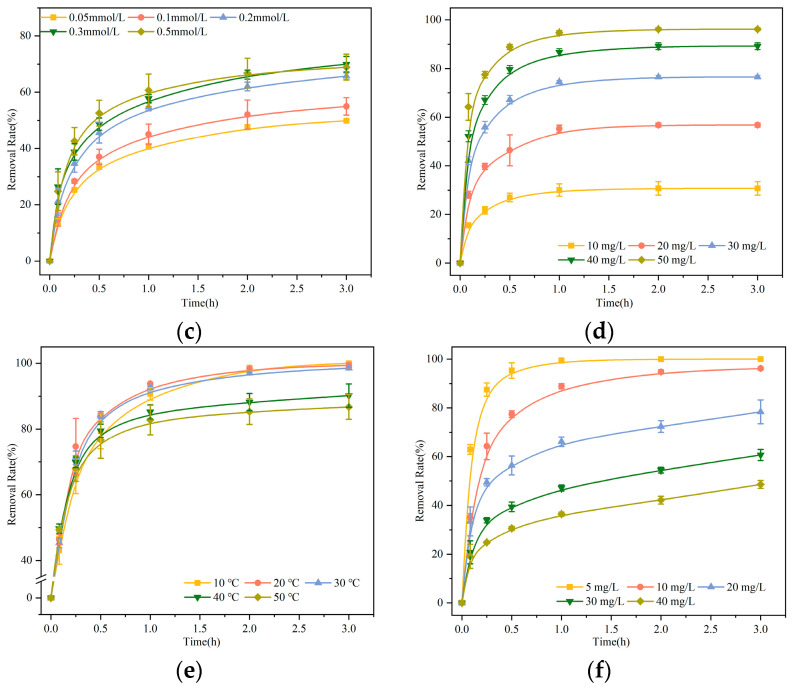
Screening of various mediators, different lowercase letters in this figure indicate significant differences among treatments (*p* < 0.05). (**a**); degradation of SDZ by BMO/SYR system with different conditions, including initial pH (**b**), SYR concentration (**c**), BMO concentration (**d**), temperature (**e**), and SDZ concentration (**f**).

BMO coupled with nine different redox mediators was evaluated based on SDZ transformation efficiency to screen for the optimal mediator. A significant reduction in SDZ concentration was observed for the BMO/SYR and BMO/acetosyringone (AS) systems compared to the other BMO/mediator combinations (Figure 2a). Since SYR and AS belong to the same category and have similar molecular structures, SYR was selected for subsequent investigations due to its higher degradation rate.

#### 2.2.1. Effect of pH on SDZ Removal

Figure 2b illustrates the effect of initial pH on SDZ removal efficiency by the BMO/SYR system. Overall, as the pH decreased from alkaline to weakly acidic conditions, the SDZ removal efficiency by the BMO/SYR system increased significantly, reaching 71.47% at pH 5.0 after 3 h of reaction. When the pH decreased to 4.0, the removal rate declined sharply to 0.89%. This trend may be attributed to variations in the speciation of SDZ and SYR, as well as changes in the surface charge of BMO in aqueous solution. The pH_zpc_ of BMO is 3.79, indicating that when the solution pH exceeds this value, the BMO surface acquires a negative charge [27]. SYR predominantly exists in its neutral form in solutions with pH < 7.3 due to its pKa value of 7.3 [28]. Thus, the weak electrostatic attraction between BMO (negatively charged) and SYR (neutral) under weakly acidic conditions may promote free radical generation, thereby improving the degradation efficiency of SDZ. In addition, SDZ is a zwitterionic compound (pKa_1_ = 2.00 and pKa_2_ = 6.48) [1], which predominantly exists as a more readily degradable neutral species within the pH range of 2.00–6.48 [29]. At pH 8.0, both SDZ and SYR exist as anion species, while the negative surface charge of BMO is further intensified. Consequently, electrostatic repulsion dominates interactions among the three components, resulting in a significant reduction in the SDZ degradation rate to just 14.61%. Beyond electrostatic effects, pH also influences the oxidizing capacity of manganese oxides and the conversion kinetics of SYR to free radicals. Previous studies have demonstrated that H^+^ ions enhance the redox potential of manganese oxides and accelerate their redox reactions with tetracycline [30]. The sharp decline in the degradation rate at pH 4.0 might be attributed to BMO dissolution induced by excessively low pH [31]. Therefore, pH 5.0 was selected as the optimal condition for the follow-up study.

#### 2.2.2. Effect of SYR Levels on SDZ Removal

Figure 2c shows the effect of the SYR concentration on the removal of SDZ by the BMO/SYR system. The removal rate of SDZ by the system was positively correlated with the SYR level within the low concentration range (0.05–0.3 mmol/L). At 0.3 mmol/L SYR, the removal rate (69.99%) was 20.11% higher than that at 0.05 mmol/L SYR. Further elevating the SYR concentration to 0.5 mmol/L produced no statistically significant difference in pollutant removal efficiency relative to the 0.3 mmol/L SYR group. This observation is analogous to the substrate inhibition phenomenon in enzyme catalysis processes [29]. At low SYR concentrations, the mediator had greater access to BMO surfaces, facilitating free radical generation that contributes to SDZ degradation. Once SYR concentration reached a threshold level, the active sites on the BMO surface might become saturated with the mediator. Free radical generation consequently reached a plateau [29]. Many previous studies have also reported that laccase-mediated degradation of antibiotics, e.g., sulfamethoxazole [13], tetracycline [32], and norfloxacin [33], initially increases with mediator concentration before plateauing. Moreover, at a high level of SYR, the probability of self-coupling reactions among intermediate benzoquinone free radicals generated in the system increased, consuming the free radicals that would otherwise react with SDZ [34,35,36]. So, the SYR level was supposed to be 0.3 mmol/L, balancing SDZ degradation efficiency and mediator dosage.

#### 2.2.3. Effect of BMO Levels on SDZ Removal

The concentration of BMO also significantly influenced the removal efficiency of SDZ in the system. As shown in Figure 2d, the removal rate of SDZ increased with the increase in BMO dosage. The maximum removal rate of 96.16% was achieved at 3 h with a BMO dosage of 50 mg/L, which was 3.13 times higher than that at 10 mg/L. A higher BMO level remarkably accelerated the initial reaction rate during the early stage of the reaction. At the same SYR concentration, a higher BMO dosage provided more redox-active Mn species and defect-related surface sites [16,36,37], which favored the activation of SYR and the subsequent transformation of SDZ in the BMO/SYR system. Similar dosage-dependent behavior has been reported on the removal of methyl blue from coffee grounds modified by sodium hydroxide, which was attributed to the greater availability of active sites on the surface [38]. Since 50 mg/L BMO in the system could almost completely degrade all SDZ, the BMO concentration was fixed at 50 mg/L.

#### 2.2.4. Effect of Temperature and SDZ Concentration on SDZ Removal

The effect of temperature on SDZ degradation by the BMO/SYR system is shown in Figure 2e. The removal rate of SDZ showed no significant variation as the temperature increased from 10 to 20 °C, with SDZ achieving nearly complete degradation (99.67%). When the temperature further increased to 40 or even 50 °C, SDZ removal efficiency decreased significantly, which was 90.15% and 86.72%, respectively. We speculate that the declined degradation efficiency at high temperatures originates from the poor thermal stability of SYR-derived phenoxyl radicals based on previous studies. Elevated temperatures facilitate radical dimerization, which consume most reactive radicals before they react with SDZ. Additionally, the intermediate DMBQ decomposes rapidly under heating and further weakens pollutant removal [39]. Generally, enzyme catalytic activity is highly temperature-dependent [40]. For example, the removal rate of tetracycline by a horseradish peroxidase/mediator was higher at 37 °C [20], and the optimal degradation of isoproturon by a laccase/hydroxybenzotriazole system was achieved at 50 °C [41]. Both systems operate optimally above room temperature and require thermal input. The high SDZ degradation efficiency of the BMO/SYR system at low to room temperature highlights its potential applicability for the remediation of groundwater and lake ecosystems under natural temperature conditions.

The degradation performance of the BMO/SYR system at various initial SDZ levels is shown in Figure 2f. The SDZ removal efficiency showed a negative correlation with its initial concentration. With the initial concentration rising from 5 to 40 mg/L, the removal rate decreased from nearly 100% to approximately 50% after 3 h of reaction. Consistent with previous studies, elevated substrate concentrations tend to occupy and saturate redox-active sites on BMO, thereby inducing substrate-inhibition-like effects [16]. What is more, the formation of excess intermediate products led to competitive adsorption with the parent compound for active sites [37]. Compared with other removal systems (such as MnO_2_ and HRP/PDS/H_2_O_2_), the BMO/SYR system exhibited a certain competitive SDZ removal performance under the tested conditions (Table S4).

### 2.3. Influence of Humic Acid and Various Ions on SDZ Removal

**Figure 3:**
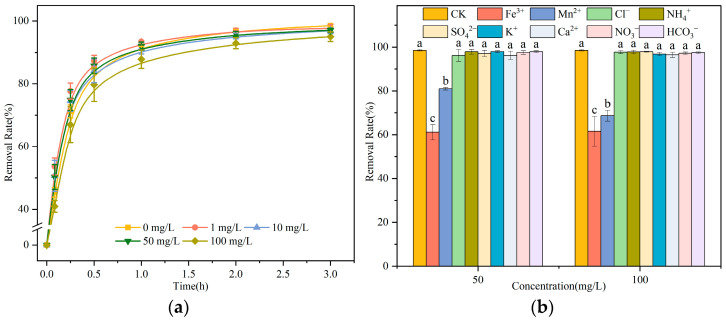
Influence of humic acid (**a**) and various ions (**b**) on SDZ removal in BMO/SYR system. Different lowercase letters in (**b**) indicate significant differences among treatments at the same ion concentration (*p* < 0.05).

The influence of humic acid (HA) on SDZ removal by the BMO/SYR system is displayed in Figure 3a. Within the initial 5 min, the addition of 1 mg/L HA increased the SDZ degradation rate by 8.27%, whereas 100 mg/L HA slightly reduced it by 4.50%. As the reaction progressed, the difference induced by varying HA concentrations diminished and eventually disappeared after 3 h. This differed from previous studies, where 50 mg/L HA reduced the SDZ degradation rate by about 20% in HRP coupled with a 1-hydroxybenzotriazole system [29]. HA exhibited a more pronounced inhibition of the HRP/persulfate system, resulting in a 40% reduction in SDZ degradation at 5 mg/L [2]. The insensitivity of the BMO/SYR system to HA could be attributed to a balance between two competing effects: the enhanced degradation resulting from HA oxidation by BMO to generate reactive species [42] and the inhibitory effect of HA on the competitive interaction with SDZ [2].

The influence of various common environmental ions and their strength on SDZ conversion is shown in Figure 3b. Most ions, such as Ca^2+^, K^+^, Cl^−^, and NO_3_^−^, had no significant effect on SDZ degradation, except Fe^3+^ and Mn^2+^. At 50 mg/L Fe^3+^, the SDZ degradation rate was inhibited by 37.40%, and increasing the Fe^3+^ concentration to 100 mg/L did not alter the inhibition magnitude. Under weak acid conditions, Fe^3+^ readily hydrolyzes to ferric hydroxide complexes [43], which adsorb onto BMO surfaces, suppress the oxidative capacity of Mn [44] and further decrease SDZ removal efficiency. For Mn^2+^, the SDZ removal was reduced by 17.53% and 36.99% at 50 and 100 mg/L, respectively. The presence of exogenous Mn^2+^ might shift the redox equilibrium of BMO (MnO_2_(s) + 4H^+^ + 2e^−^ → Mn^2+^+ 2H_2_O), thereby suppressing subsequent oxidative reactions of BMO [37]. This was similar to the substrate inhibition principle observed in enzyme-catalyzed reactions [45]. Moreover, Mn^2+^ might be adsorbed onto the BMO surface and occupied redox-active sites, thereby further hindering SDZ transformation [37].

### 2.4. Transformation Products and Pathway

To further elucidate the transformation pathway of SDZ in the BMO/SYR system, ultra-high-performance liquid chromatography–tandem mass spectrometry (UHPLC-MS/MS) was employed to identify the intermediates formed during the degradation process. Based on the accurate mass measurements and MS/MS fragmentation patterns, four SDZ transformation products (TP1–TP4) and one coupling product (SDZ-DMBQ) were tentatively identified, as summarized in Table S2. The extracted ion chromatograms, together with the full-scan MS and MS/MS spectra of the parent compound and the identified products, are provided in Figures S6–S9.

The retention time of the parent compound SDZ was 4.54 min, with *m*/*z* ratios of 251.0593 and 249.0450 in positive and negative modes, respectively. TP1 was detected in positive ion mode with a protonated ion at *m*/*z* 96.0561. It was tentatively assigned the molecular formula C_4_H_5_N_3_, corresponding to the 2-aminopyrimidine moiety released from the SDZ skeleton. The formation of TP1 indicates that cleavage occurred adjacent to the sulfonamide linkage, most likely via cleavage of the S8-N9 sulfonamide bond. This scission process constitutes Pathway I of SDZ degradation. In contrast, the complementary aromatic fragment (proposed product 1) was not directly identified in the reaction system. This absence is most likely due to the failure of this transient intermediate to accumulate to detectable concentrations, coupled with its rapid secondary transformation. Previous studies have shown that cleavage of the S8–N9 bond is often accompanied by subsequent hydroxylation of the resulting aromatic moiety, which likely accounts for the lack of observable proposed product 1 in the present analysis [2,18]. In addition to the sulfonamide S–N bridge [46], SDZ harbors multiple susceptible sites for bond cleavage and oxidative modification, including the C2–S8 arylsulfonyl bond [47], the amino group at the N7 position [18], and various covalent bonds within the pyrimidine ring [48]. By contrast, only TP1 was identified as a direct cleavage-derived product in the BMO/SYR system, whereas other cleavage products commonly reported in bacterial [46], enzymatic [49], and electrochemical oxidation [48] systems were not detected in this work.

Pathway II represented the Smiles-rearrangement-mediated transformation of SDZ. Desulfonylation is widely recognized as a critical step in the degradation of sulfonamide antibiotics. However, direct oxidative attack on the sulfonamide moiety is often hindered by the steric hindrance surrounding the central sulfonyl group [19]. The Smiles rearrangement circumvents this limitation by rearranging the molecular configuration of SDZ, which exposes the sulfonamide-associated groups and renders them more susceptible to subsequent transformation reactions [29]. As depicted in Figure S10, this intramolecular rearrangement is initiated by cleavage of the C2-S8 bond, followed by the formation of a new N–C2 bond. Subsequent dehydrogenation and formation of the N9=C12 double bond completed the intramolecular rearrangement, yielding a stable Smiles-rearranged SDZ intermediate. In the proposed pathway, this rearranged intermediate could further undergo hydroxylation to generate proposed product 2 (possible but not detected). Following the extrusion of SO_3_, TP2 was formed. TP2 may then undergo further oxidation, first via carbonylation to yield TP4, followed by hydroxylation of TP4 to produce TP3. This Smiles-rearrangement-driven degradation cascade is consistent with findings from previous studies on SDZ transformation, such as the HRP-catalyzed SDZ degradation process [29].

**Figure 4:**
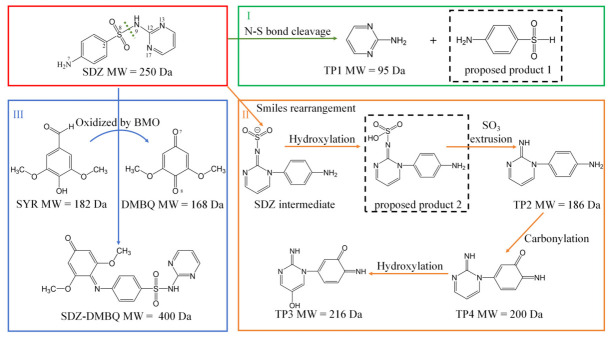
The possible degradation pathways of SDZ in the BMO/SYR system. (**I**) N–S bond cleavage; (**II**) Smiles rearrangement followed by SO_3_ extrusion and further oxidation; and (**III**) oxidation of SYR to DMBQ and subsequent coupling with SDZ. Dashed boxes indicate proposed products that were not detected.

Pathway III corresponds to the covalent coupling reaction between SDZ and oxidation products derived from the redox mediator. Previous studies have reported that SYR can be first oxidized by enzymatic or chemical catalysts to phenoxy radicals, which may subsequently be converted to 2,6-dimethoxy-1,4-benzoquinone (DMBQ) [19]. In the BMO/SYR system, the resulting DMBQ can react further with SDZ to form the coupling product SDZ-DMBQ. Distinct from the bond cleavage and Smiles rearrangement pathways, this coupling route represents a unique mediator-involved transformation mechanism, in which the redox mediator itself is incorporated into the final product structure. Accordingly, DMBQ-mediated coupling is regarded as a notable transformation pathway for SDZ in the BMO/SYR system. Overall, the putative major transformation pathways of SDZ in the BMO/SYR system included cleavage, Smiles rearrangement followed by SO_3_ extrusion and further oxidation, and covalent coupling with SYR-derived DMBQ, as shown in Figure 4.

### 2.5. Theoretical Calculation

**Figure 5:**
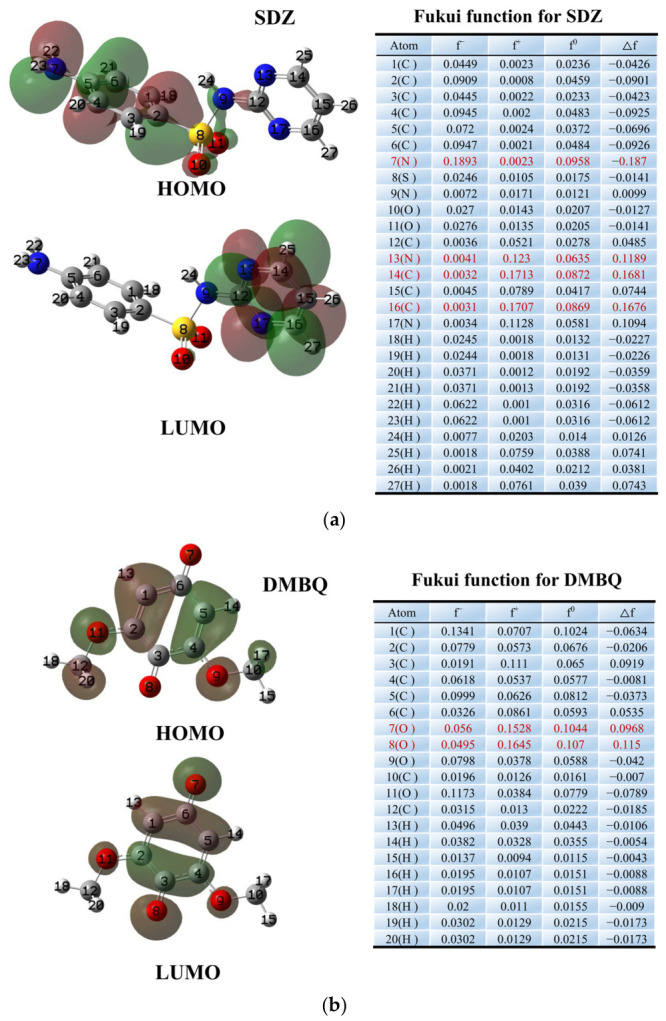
The HOMO, LUMO and Fukui functions parameters of SDZ (**a**) and DMBQ (**b**).

Figure 5 displays the frontier molecular orbital distributions of SDZ. In the HOMO plot, relatively large orbital contributions were mainly observed around the N7 site on the aniline moiety and the sulfonamide group, suggesting that these regions may have stronger electron-donating ability and are therefore more susceptible to oxidation or attack by electrophilic species. In contrast, the LUMO distribution was predominantly localized on the pyrimidine ring, indicating that this region may serve as an electron-accepting site and thus be more susceptible to nucleophilic attack. These frontier orbital distributions suggest that the aniline/sulfonamide region and the pyrimidine ring are potential reactive domains of SDZ, which is consistent with the proposed reaction pathway.

Fukui function calculation results are presented in Figure 5. For sites susceptible to electrophilic attack, as characterized by f^−^ values, the f^−^ value of N7 was 0.1893, while C6, C4, and C2 also exhibited relatively high f^−^ values, indicating that these sites possessed strong electron-donating ability and were prone to attack by electrophilic species. For sites susceptible to nucleophilic attack, as characterized by f^+^ values, C14, C16, N13, and N17 exhibited the highest f^+^ values, indicating that they were potential electrophilic sites. The Δf value of N7 was the most negative (−0.187), further indicating its strong nucleophilic tendency. The Δf values of C14 (0.1681) and C16 (0.1676) were considerably positive, indicating their strong electrophilic tendency. Fukui function analysis revealed that the pyrimidine ring, benzene ring, and amino group of SDZ were all potential reactive sites, providing theoretical support for their possible involvement in the proposed reaction pathway. For DMBQ, the carbonyl region showed pronounced electrophilic character, as reflected by the markedly high f^+^ values and positive Δf values of O7 and O8. This result suggests that the carbonyl groups of DMBQ were highly reactive and could participate in the reaction with the amino group of SDZ, supporting the occurrence of the DMBQ–SDZ coupling reaction in Pathway III.

### 2.6. Assessment of Byproduct Toxicity

**Figure 6:**
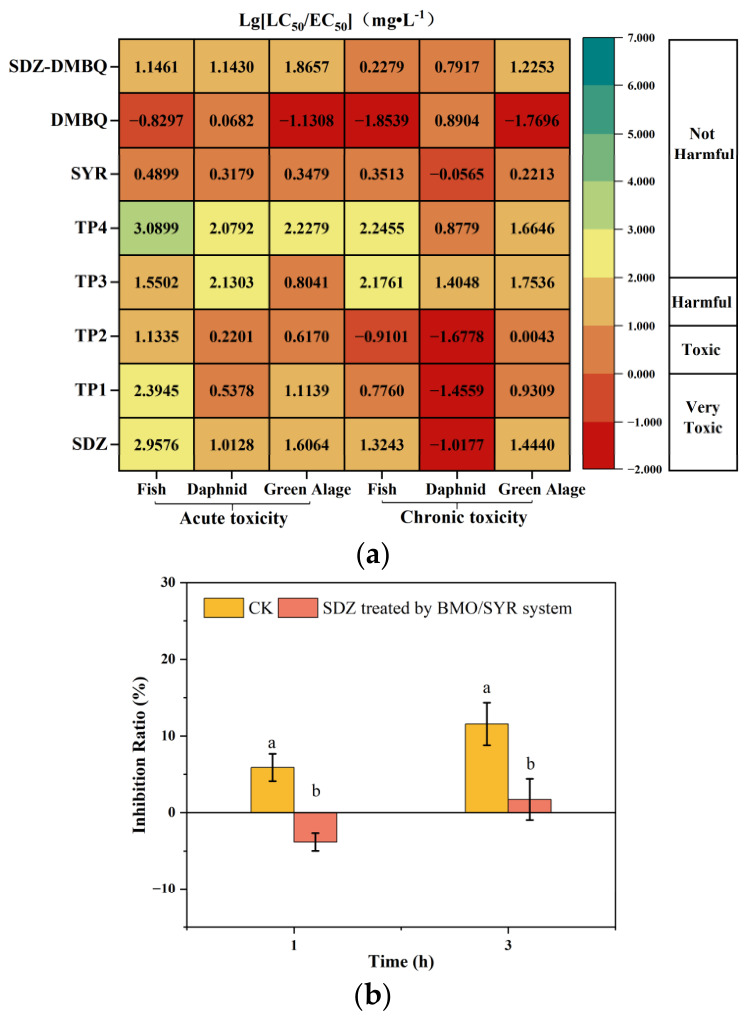
Assessment of the toxicity of SDZ and its byproducts by ECOSAR (**a**) and their inhibition on growth of *Escherichia coli* (**b**). Different lowercase letters in (**b**) indicate significant differences between treatments at the same incubation time (*p* < 0.05).

The acute and chronic toxicity of transformation products generated in the BMO/SYR system was evaluated via the ECOSAR software to fish, daphnia, and green algae, with results presented in Figure 6a. In general, the deeper degradation products TP3 and TP4 displayed remarkably weaker acute and chronic toxicity than the parent SDZ, falling into the non-harmful grade. In contrast, several early-stage transformation intermediates (TP1 and TP2) showed moderately elevated toxicity relative to SDZ with elevated chronic toxicity specifically against fish and daphnia.

Among all tested compounds, the terminal oxidation product of the SYR mediator, DMBQ, exhibited the most pronounced predicted toxicity. However, DMBQ is a reactive quinone-type intermediate and is not expected to remain completely stable in the reaction system. Previous studies have reported that DMBQ generated from SYR oxidation can further participate in coupling reactions with sulfonamide-derived intermediates or undergo self-coupling reactions, suggesting that excess DMBQ may be further transformed during the reaction [19]. To further evaluate the residual toxicity of the treated solution, a bacterial growth inhibition assay was performed using *Escherichia coli*. As shown in Figure 6b, the growth inhibition rate of *Escherichia coli* induced by SDZ samples after degradation in the BMO/SYR system was significantly reduced by 9.85% compared with untreated SDZ. Therefore, although ECOSAR prediction indicated that some individual intermediates may still possess relatively high biological toxicity, the experimental toxicity assay demonstrated that the overall residual toxicity of the reaction solution decreased after BMO/SYR treatment. These results suggest that the BMO/SYR system exerted a beneficial effect on SDZ detoxification, while the environmental behavior and long-term ecological risks of quinone-type intermediates should be further evaluated.

### 2.7. Mechanism of SDZ Transformation by the BMO/SYR System

**Figure 7:**
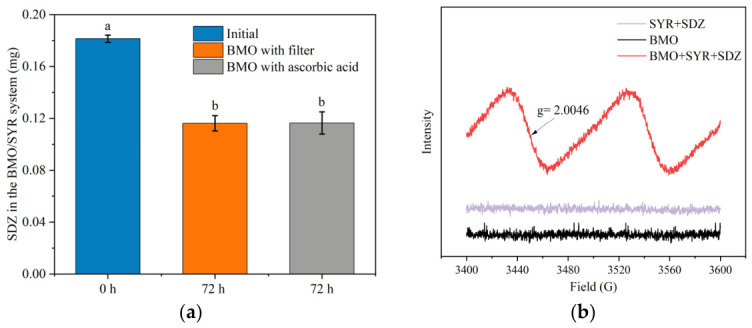

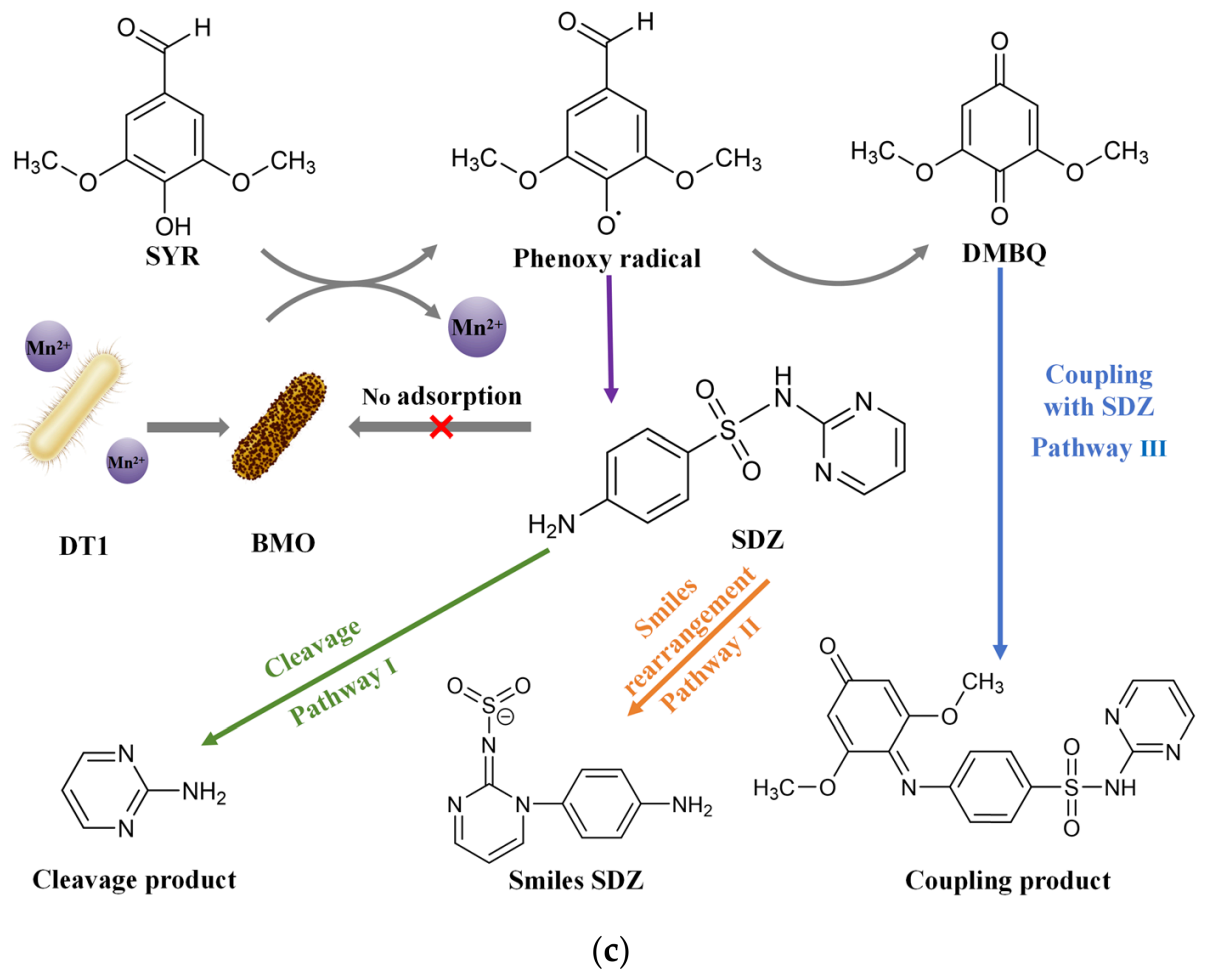
The adsorption of SDZ by BMO (**a**); the EPR spectra obtained from SYR/SDZ and BMO/SYR/SDZ with the existence of DMPO (**b**); conceptual scheme of SDZ removal by BMO/SYR system (**c**). Different lowercase letters in the inset of (**a**) indicate significant differences among treatments (*p* < 0.05).

Previous studies have reported that BMO removes 17β-estradiol [11], sulfamethoxazole [14], and bisphenol A [50] via both adsorption and oxidative degradation. Thus, this study further evaluated the contribution of BMO-mediated adsorption to SDZ removal in the BMO/SYR system. As shown in Figure 7a, no significant difference in SDZ concentration was observed between the filtrate and the solution after BMO reductive dissolution with ascorbic acid, indicating that the adsorption contribution of BMO to SDZ removal was negligible under the tested conditions. Moreover, the oxidative activity of BMO alone (50 mg/L) toward SDZ over 3 h was nearly negligible. As shown in Figure 7b, a distinct EPR signal was detected in the BMO/SYR/SDZ group, which was absent in the control groups (SYR/SDZ and BMO alone). This indicated that the signal likely originated from BMO-mediated oxidation of SYR. The g-value of this signal was determined to be 2.0046, which was consistent with the typical EPR signature of phenoxyl radicals reported in previous studies [51,52]. Previous studies have shown that syringaldehyde and related syringyl-type phenolic mediators can be oxidized by mild oxidants, including laccase and MnO_2_, to generate phenoxy radicals, which can further form quinone-type products such as DMBQ [19,53]. DMBQ-involved coupling pathways have also been reported in laccase–syringaldehyde and related syringyl-type mediator systems for sulfadiazine transformation [18,54]. Therefore, combined with the EPR signal and LC-MS-identified DMBQ-related products (Figure S7) in this study, BMO-mediated oxidation of SYR was proposed to generate SYR-derived phenoxyl radical-like species and DMBQ, which subsequently participated in SDZ transformation. Based on these results, the possible mechanism of SDZ degradation in the BMO/SYR system was proposed. As shown in Figure 7c, SYR was first oxidized to phenoxyl radicals by BMO. These radicals then participated in the cleavage and Smiles rearrangement of SDZ (Pathways I and II). Afterwards, SYR-derived phenoxyl radicals were further transformed into DMBQ, which couples with SDZ to proceed via Pathway III.

### 2.8. Potential Applicability of the BMO/SYR System

**Figure 8:**
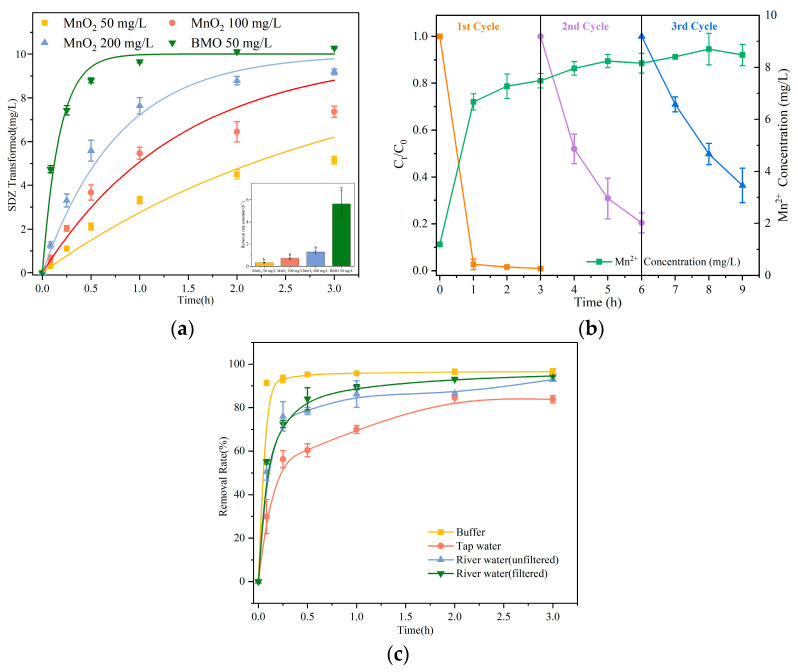
Comparison of the ability of BMO and CMO to mediate SYR degradation of SDZ (**a**); reusability of BMO in the BMO/SYR system for SDZ degradation, where C_t_/C_0_ represents the ratio of residual SDZ concentration to initial SDZ concentration (**b**); effect of different water matrices on SDZ by BMO/SYR systems (**c**). Different lowercase letters in the inset of (**a**) indicate significant differences among treatments (*p* < 0.05).

The SDZ degradation efficiency by MnO_2_ and BMO-mediated SYR was compared, as shown in Figure 8a. SDZ degradation in both systems followed pseudo-first-order kinetics, with R^2^ values ranging from 0.9653 to 0.9946. As the concentration of CMO increased from 50 mg/L to 200 mg/L, the transformation rate constant (K) increased from 0.32 to 1.28 h^−1^, which remained substantially lower than that in the BMO system. The K values of the 50 mg/L BMO group were 17.47, 7.96, and 4.41 times higher than those of the three CMO groups, respectively. This observation is consistent with previous findings, which showed that BMO-mediated peroxymonosulfate activation for phenol and tetracycline degradation resulted in a reaction rate 3 times higher than that with MnO_2_ [16]. A comparison of the XPS spectra of BMO and CMO showed that the manganese oxidation state distribution of CMO was similar to that of BMO (Figure 1b and Figure S11a), while the proportion of surface defect-related/non-lattice oxygen was slightly higher than that of BMO (Figure 1c and Figure S11b). XRD analysis showed that CMO had a poorly crystalline structure but exhibited more obvious diffraction characteristics than BMO (Figure S11c). Although CMO possesses detectable oxygen defects, the catalytic performance is governed by the comprehensive properties of materials. Compared with BMO, CMO exhibits higher crystallinity. This structural characteristic impedes interfacial electron transfer, counteracts the promotional effect induced by oxygen defects, and results in a weaker SYR activation capacity [55]. These structural differences may contribute to the lower SDZ transformation efficiency of the CMO/SYR system compared with the BMO/SYR system. IM et al. also reported that *Pseudomonas putida*-derived BMO achieved a five-fold-higher BPA degradation efficiency compared with CMO [8]. Overall, the higher transformation efficiency of BMO suggests that the BMO/SYR system has potential advantages for SYR-mediated SDZ removal and provides a basis for further exploring its application potential in pollutant treatment.

The reusability of BMO is shown in Figure 8b. The final SDZ removal efficiency decreased by approximately 20% with each reuse of BMO. The final removal efficiency decreased from nearly 100% in the first cycle to approximately 63% after the third cycle. The high-valence Mn species in BMO could be reduced during repeated use, releasing Mn^2+^. This led to a gradual reduction of the effective oxidative sites, thereby weakening the pollutant transformation efficiency of the system [14]. As shown in Figure 8b, the content of Mn^2+^ gradually increased with the degradation of SDZ. In addition, XPS spectra (Figure S12) showed that the abundance of Mn(II)/Mn(III)/Mn(IV) in the used BMO was lower than that in the fresh BMO, and the atomic ratio of Mn(III)/Mn(IV) decreased. These were all possible reasons for the decline in SDZ transformation efficiency after multiple cycles. The release of Mn^2+^ may pose potential secondary ecological risks, which may not be conducive to the application of this method. Previous investigations have confirmed that Mn^2+^ leached from BMO can undergo microbial oxidation to form Mn(IV), indicating the feasibility of microbially mediated BMO regeneration and manganese cycling [50]. Therefore, Mn^2+^ recovery, microbial reoxidation, and BMO regeneration may be considered in future studies to improve the cyclic use and potential applicability of the BMO/SYR system.

The application potential of the BMO/SYR system in real water matrices was evaluated, with results illustrated in Figure 8c. The removal rates of SDZ in filtered river water, unfiltered river water, and tap water were reduced by 11.24%, 16.79%, and 34.89% within the initial 30 min relative to the buffer control, but these differences narrowed to 2.06%, 3.70%, and 12.78% after 3 h of reaction. Generally, impurities present in actual matrices, such as suspended solids, ammonia nitrogen, chemical oxygen demand, dissolved organic matter, and coexisting ions, may interfere with the oxidative activity of manganese oxide-based systems [56,57]. As mentioned earlier, the BMO/SYR system exhibited high tolerance to most common environmental ions, which might also explain the narrowed inhibition in the later stage of degradation in actual matrices. Overall, these results suggested that the BMO/SYR system has potential applicability in actual water matrices. Similar sustainable, nature-based water treatment approaches validated at a semi-industrial scale have been recently reported, such as the cactus-leaf natural coagulant system for real turbid drinking water purification, which also achieves efficient removal of suspended solids and pathogenic microorganisms without toxic chemical additives [58].

## 3. Materials and Methods

### 3.1. Chemicals and Culture Medium

Analytical-grade sulfadiazine (SDZ), leucoberbelin blue I (LBB), and syringaldehyde (SYR) were obtained from Aladdin Biochemical Technology Co., Ltd. (Shanghai, China). High-performance liquid chromatography-grade acetonitrile and formic acid were purchased from Fisher Scientific (Waltham, MA, USA). The chemical manganese oxide (CMO) was synthesized according to a previous method with detailed procedures provided in the Supporting Information (SI) [59]. The experimental strain, *Stenotrophomonas maltophilia* DT1, was isolated from tetracycline-contaminated soil (GenBank accession number: MLJK00000000) and harbors a multicopper oxidase gene [3]. The Lysogeny broth (LB) medium was used to seed activation, and PCYM medium was employed for BMO production [60].

### 3.2. Formation and Characterization of BMO

Strain *S. maltophilia* DT1 was inoculated into LB medium and incubated at 30 °C on a shaker at 150 rpm. The logarithmic growth phase cells were washed twice with phosphate buffer solution (PBS). Then, the activated cells were transferred to PCYM medium containing 200 mg/L Mn^2+^ with an initial OD_600_ of 0.4. The culture was incubated at 30 °C with shaking at 150 rpm for 7 days. Finally, the culture was centrifuged at 10,000 rpm for 15 min and washed three times with PBS to collect BMO samples. The concentration of BMO was quantified using the LBB method, with detailed procedures provided in the SI. The morphology, elemental composition, and oxidation states of BMO were characterized using scanning electron microscopy coupled with energy-dispersive spectroscopy (SEM–EDS mapping, TESCAN MIRA LMS, Brno, Czech Republic), X-ray photoelectron spectroscopy (XPS, Thermo Scientific K-Alpha, Waltham, MA, USA), and X-ray diffraction (XRD, Rigaku Smartlab SE, Tokyo, Japan) based on previously reported methodologies [16,61]. Electron paramagnetic resonance (EPR, Bruker EMX plus 6/1, Rheinstetten, Germany) was employed to characterize the defect-related signal (g ≈ 2.0) of the samples [62].

### 3.3. Batch Experiments

All incubation experiments were performed in 50 mL amber serum vials. An amount of 20 mL of a 0.2 mol L^−1^ (pH 4.0–8.0) buffer solution was added to each reactor, which contained redox mediators (0.05–0.5 mmol/L), BMO (10–50 mg/L) and SDZ (5–40 mg/L), which were then incubated at various temperatures (10–50 °C). Then, 1 mL reaction samples were filtered and the residual concentration of SDZ was measured at 5, 15, 30, 60, 120, and 180 min. Similar batches were conducted under optimized conditions to investigate the effects of humic acid (1–100 mg/L) and various ions (50 and 100 mg/L of Na^+^, K^+^, Ca^2+^, Mn^2+^, Fe^3+^, Cl^−^, NO_3_^−^, SO_4_^2−^, and HCO_3_^−^). A comparative study of SDZ removal efficiency between BMO and chemically synthesized manganese oxide (CMO, 50–200 mg/L) was conducted, and the characterization results of CMO, including XPS and XRD, can be found in the Supporting Information (SI). Furthermore, the reusability of BMO and its practical applicability in real aqueous matrices were evaluated. For BMO reusability tests, the aforementioned optimized reaction conditions were adopted, with the SDZ concentration set at 10 mg/L. The concentration of dissolved Mn^2+^ released during the cycling experiments was determined according to the method described by Yu et al. [63]. During the reaction, amber serum vials were placed in a water bath for incubation. Manual shaking was performed every 15 min and prior to sampling to ensure homogeneous mixing of the reaction system. For evaluating the practical application of the BMO/mediator degradation system, PBS buffer was replaced with tap water and river water, and the pH of each matrix was adjusted to 5.0. All experiments were performed in triplicate.

### 3.4. Determination of SDZ Concentration

High-performance liquid chromatography (HPLC, UltiMate 3000, Thermo, Waltham, MA, USA) equipped with a Waters XBridge^TM^ C18 column (4.6 × 150 mm, 5 µm) was used to quantify SDZ concentration. SDZ was subjected to gradient elution using a mobile phase consisting of 0.1% formic acid in water (A) and acetonitrile (B) with a flow rate of 1.4 mL/min at 260 nm. The gradient elution procedure was as follows: 0–3.5 min, 20% B; 3.5–5.5 min, 90% B; 5.5–8.5 min, 20% B. Representative HPLC chromatograms used for SDZ quantification are provided in Figure S13.

### 3.5. Identification of SDZ Degradation Products

Post-reaction samples were purified and enriched using Oasis HLB cartridges (6 cc/500 mg, Waters, Milford, MA, USA) [2]. The treated samples (5 μL) were separated using an UltiMate 3000 UHPLC system equipped with an ACQUITY UPLC^TM^ BEH C18 column (2.1 × 100 mm, 1.7 μm, Waters) and then introduced into a Q-Exactive Orbitrap tandem mass spectrometer (UHPLC-MS/MS, Thermo Scientific) equipped with an ESI source. The mobile phase consisted of a 0.1% formic acid solution (A) and acetonitrile (B), with a flow rate of 0.2 mL min^−1^. The gradient elution program was as follows: 0–9 min, 10–90% B; 9–12 min, 90% B; 12–15 min, 90–10% B; and 15–16 min, 10% B. The Q-Exactive mass spectrometer was operated in full MS/ddMS^2^ acquisition mode under both positive and negative ionization modes, with a scan range of *m*/*z* 50–750. The ion source parameters were as follows: gas temperature, 350 °C; capillary voltage, 3500 V; fragmentor voltage, 175 V; skimmer voltage, 65 V; and oct RFV, 750 V. HCD fragmentation was carried out using stepped normalized collision energy (NCE) values of 10 and 15. An SDZ-only control was analyzed under the same UHPLC-ESI-MS/MS conditions to assist in distinguishing reaction-related product signals from possible source-related signals.

### 3.6. Toxicity Assessment

The ECOSAR software (EPA, version 2.2; U.S. Environmental Protection Agency, Washington, DC, USA) was used to evaluate the acute and chronic toxicity of the byproducts toward representative aquatic organisms, including fish, daphnia, and green algae, following the protocol described previously [64]. In addition, *Escherichia coli* ATCC 25922 was used as an indicator organism to evaluate the potential environmental toxicity of the transformation products. Sterile tubes were each loaded with 8 mL of sterilized LB medium and 1 mL of *E. coli* culture (logarithmic growth phase, OD_600_ adjusted to 0.5). Subsequently, 1 mL of sterilized water, 1 mL of BMO/SYR-treated SDZ sample, and 1 mL of untreated SDZ sample were added to the three respective tubes. The tubes were incubated at 37 °C for 1 h and 3 h, respectively, after which aliquots were sampled to measure the OD_600_ values. The growth inhibition rate was calculated according to Equation (1).

(1) $$Growth\;inhibition\;rate\left(\%\right)=\frac{{OD}_{water}-{OD}_{sample}}{{OD}_{water}}$$

### 3.7. Free Radical Trapping in the System

The electron paramagnetic resonance (EPR) technique (Bruker A300 EMX PLUS, Rheinstetten, Germany) was used to detect free radicals generated in the following groups: SYR + SDZ and BMO + SYR + SDZ. The concentrations of reactants in the reaction mixtures were as follows: 0.3 mmol/L SYR, 50 mg/L BMO, and 10 mg/L SDZ. The 5,5-Dimethyl-1-pyrroline N-oxide (DMPO) was added at a concentration of 200 mmol/L to ensure an excess. The instrument parameters were set as follows: center field = 3510 G; sweep width = 100 G [65]. Measurements were conducted 10 min after the reaction was initiated.

### 3.8. Theoretical Calculations

Density functional theory (DFT) calculations were performed to identify reactive sites on SDZ and DMBQ and to evaluate their propensity for bond cleavage. All calculations were carried out using Gaussian 09 at the B3LYP/6-311+G(d,p) level with the SMD implicit solvation model (water). The molecular geometry of SDZ was fully optimized without symmetry constraints, and vibrational frequency analysis was performed to confirm that the optimized structures corresponded to true energy minima. Frontier molecular orbital (FMO) analysis was employed to determine the energy levels and spatial distributions of the highest occupied molecular orbital (HOMO) and lowest unoccupied molecular orbital (LUMO). Fukui functions and condensed Fukui indices were subsequently calculated based on Mulliken population analysis using the Multiwfn program (version 3.8) [66,67], enabling the identification of electrophilic and nucleophilic attack sites in SDZ and DMBQ.

## 4. Conclusions

This study synthesized BMO by *Stenotrophomonas maltophilia* DT1, which contained mixed-valence Mn species, including Mn(II), Mn(III), and Mn(IV), together with defect-related oxygen species and a poorly crystalline structure. Under the optimized conditions of pH 5.0, 0.3 mmol/L SYR, 50 mg/L BMO, and 10–20 °C, the BMO/SYR system achieved 99.67% SDZ removal within 3 h. The system showed good tolerance to humic acid and most common environmental ions, except Fe^3+^ and Mn^2+^. LC-MS analysis identified five possible transformation products, and three main transformation pathways were proposed, including SDZ cleavage, Smiles rearrangement, and DMBQ-involved coupling. EPR analysis qualitatively confirmed that BMO could mediate SYR to form the phenoxyl radical-like species, which may play an important role in SDZ transformation. Toxicity assessment indicated that the overall toxicity decreased after BMO/SYR treatment. Compared with CMO, BMO showed 4.41–17.47 times higher SYR-mediated SDZ transformation efficiency. Tests in real water matrices suggested that the BMO/SYR system may have potential applicability in aqueous environments. These findings may provide an efficient and environmentally friendly technology for SDZ pollution remediation and offer potential support for the removal of refractory pollutants using biogenic manganese oxide-mediated systems. For the Mn(II) release in cyclic use, future work can focus on the system by BMO immobilization and Mn^2+^ recovery strategies to further improve its engineering application potential.

## Data Availability

The original contributions presented in this study are included in the article. Further inquiries can be directed to the corresponding authors.

## Supplementary Materials

The following supporting information can be downloaded at https://www.mdpi.com/article/10.3390/molecules31142484/s1, Table S1. Abbreviations of various redox mediators; Table S2. Characteristics of the parent compound and degradation products by the BMO/SYR system; Table S3. The properties of this river water; Table S4. Comparison of SDZ removal efficiency by different treatment systems; Figure S1. The initial (a) and 7th day (b) status of DT1 in PCYM medium; the BMO produced by DT1 caused LBB to change from transparent to blue (c); Figure S2. The EDS results of BMO; Figure S3. The mapping results of BMO; Figure S4. Atomic ordering of SDZ; Figure S5. Atomic ordering of DMBQ; Figure S6. Total ion chromatograms of (a) SDZ, (b) TP1, (c) TP2, (d) TP3, (e) TP4, (f) DMBQ, (g) SYR, and (h) SDZ-DMBQ; Figure S7. Mass spectrum of (a) SDZ, (b) TP1, (c) TP2, (d) TP3, (e) TP4, (f) DMBQ, (g) SYR, and (h) SDZ-DMBQ; Figure S8. The observed isotopic patterns of (a) SDZ, (b) TP1, (c) TP2, (d) TP3, (e) TP4, (f) DMBQ, (g) SYR, and (h) SDZ-DMBQ; Figure S9. The MS/MS fragmentation profile and proposed fragmentation pattern of (a) SDZ, (b) TP1, (c) TP2, (d) TP3, (e) TP4, (f) DMBQ, (g) SYR, and (h) SDZ-DMBQ; Figure S10. Schematic illustration of the Smiles rearrangement process of SDZ; Figure S11. XPS spectra of Mn2p (a) and O1s (b) and XRD (c) of chemical synthesis of manganese oxide (CMO); Figure S12. The difference of XPS of BMO before (a,b) and after cycling (c,d); Figure S13. Representative HPLC chromatograms of SDZ before and after reaction in the BMO/SYR system. References [2,10,29,37,40,59,60,68,69,70,71,72] are cited in the Supplementary Materials.
