# Supplementary material for: Degradation of Sulfadiazine by Biogenic Manganese Oxides Coupled with Syringaldehyde: Performance, Mechanism, Toxicity, and Environmental Applicability
Source: Molecules. 2026 Jul 16;31(14):2484. doi: 10.3390/molecules31142484 (PMC13414250; doi:10.3390/molecules31142484)
Supplement: Supplementary file 1 [file molecules-31-02484-s001.zip › molecules-4375755-supplementary.pdf]

Supporting Information

2. Materials and Methods

2.1 Culture medium

LB medium：10 g of peptone, 5 g of yeast extract, 10 g of NaCl, and 1 L of ultrapure water.

PCYM medium：0.8 g of peptone, 0.2 g of yeast extract, 0.2 g of $MnCl_2{\cdot}4H_2O$, 0.1 g of K_2HPO_4, 0.2 g of $MgSO_4{\cdot}7H_2O$, 0.2 g of $NaNO_3$, 0.1 g of $CaCl_2$, 0.1 g of $(NH_4)_2CO_3$, and 1 L of ultrapure water [1]. Filter-sterilized Mn^{2+} was added to autoclaved PCYM medium (121 °C, 30 min).

2.2 Chemical manganese oxide synthesis

400 mL of 0.8 mol/L $KMnO_4$ (60 °C) was dropped into the 300 mL of 1.6 mol/L $MnSO_4$ (90 °C) and kept stirring continually. Then, filter the suspension and wash it several times with UP water until the pink faded. Finally, the manganese oxide was dried at 100 °C overnight [2].

2.3 BMO concentration determination

Dissolve 25 mg of LBB in 62.5 mL of 0.25% glacial acetic acid solution to obtain 0.25% LBB solution. 100 μL sample was mixed with 500 μL LBB solution, incubated at room temperature in the dark for 15 min, then the absorbance at 620 nm was measured using a spectrophotometer. The content of BMO was calculated through the curve of potassium permanganate standard solution (1-4 mg/L) [3].

2.4. Determination of the adsorption process

50 mg/L BMO and 10 mg/L SDZ were added in a 20 mL amber glass bottle. The bottles were shaken at 30 °C for 72 h. Then, one bottle was treated by membrane filtration to determine the SDZ concentration. The other sample was treated with ascorbic acid to dissolve BMO and released the adsorbed SDZ. The concentration difference between the two methods is due to adsorption [4].

2.5. Kinetics Modeling

SDZ removal by BMO/SYR system can be described by the following equation:

$$C = C_0 - C_0 \times \mathrm{e}^{-k \times T} \quad (S1)$$

C_0 is the initial concentration of SDZ (mg/L), kHB is the first-order reaction rate constant, T is the time (h)，C is the concentration change of SDZ due to the degradation (mg/L).

Table S1. Abbreviations of various redox mediators.

Name	Abbreviation
2,2′-azino-bis(3-ethylbenzothiazoline-6-sulfonic acid)	ABTS
syringaldehyde	SYR
acetosyringone	AS
1-Hydroxybenzotriazole	HBT
2,6-dimethoxyphenol	DMP
4-Coumaric acid	PCOA
vanillic aldehyde	VAN
vanillyl alcohol	VA
Violuric acid	5-HA

Table S2. Characteristics of the parent compound and degradation products by the BMO/SYR system.

Mode	RT (min)	Compound	Measured *m/z*	Ion composition	Compound formula	Intensity
Positive	4.54	SDZ	251.0593	$C_{10}H_{11}N_4O_2S^+$	$C_{10}H_{10}N_4O_2S$	1.75E+08
	1.52	TP1	96.0561	$C_4H_6N_3^+$	$C_4H_5N_3$	2.28E+06
	3.76	TP2	187.0978	$C_{10}H_{11}N_4^+$	$C_{10}H_{10}N_4$	1.12E+07
	11.12	TP3	217.0720	$C_{10}H_9O_2N_4^+$	$C_{10}H_8O_2N_4$	7.44E+07
	7.03	TP4	201.0755	$C_{10}H_9N_4O^+$	$C_{10}H_8N_4O$	6.59E+07
	7.19	DMBQ	169.0493	$C_8H_9O_4^+$	$C_8H_8O_4$	1.15E+09
Negative	9.35	SYR	181.0497	$C_9H_9O_4^-$	$C_9H_{10}O_4$	1.02E+09
	9.84	SDZ-DMBQ	399.0767	$C_{18}H_{15}N_4O_5S^-$	$C_{18}H_{16}N_4O_5S$	9.56E+07

Table S3. The properties of this river water.

Category	Value
Site	N 30° 29′ 24″, E 114° 19′ 12″
COD (mg/L)	12.61
TN (mg/L)	12.73
NH_3-N (mg/L)	2.27
TP (mg/L)	0.41
SS (mg/L)	36.12

Table S4. Comparison of SDZ removal efficiency by different treatment systems.

Treatment system	Initial SDZ concentration	Key reaction conditions	SDZ removal efficiency	Reaction time	Reference
BMO/SYR	10 mg/L	BMO 50 mg/L; SYR 0.3 mmol/L; pH 5.0; 10–20 °C	99.67%	3 h	This study
MnO_2/SYR	6 μM	MnO_2 120 μM; SYR 12 μM; pH 5–8	~20%, ~40%, ~30%, and ~20% at pH 5, 6, 7, and 8, respectively	60, 180, 250, 500 min depending on pH	[5]
UV/O_3	25 mg/L	O_3 38.3–38.4 mg/L; UV 0.30 mW/cm^2; pH 7.0	98-100%	10 min	[6]
$MnFe_2O_4$/δ-MnO_2/PMS	10 mg/L	$MnFe_2O_4$/δ-MnO_2 0.2 g/L; PMS 2.0 mM; pH 6.0; 25 °C	nearly 100%	30 min	[7]
Cu-beta zeolites/PS	50 mg/L	Cu-beta zeolites 1.0 g/L; PS 0.5 g/L; pH 7.0	90.5%	120 min	[8]
visible light irradiation/ZnFe/biochar/PMS	30 mg/L	ZnFe/biochar 0.5 g/L; PMS 5 mM; pH 6.0; visible light $\lambda \geq 400$ nm	98.8%	90 min	[9]
HRP/HBT	50 mg/L	HRP/HBT/H_2O_2 system; pH 6.0; 35 °C	94.54%	30 min	[10]
HRP/PDS/H_2O_2	10 mg/L	HRP 0.8 U/mL; PDS 2 mM; H_2O_2 0.5 mM; pH 7.0; 25 °C	62%	6 h	[11]

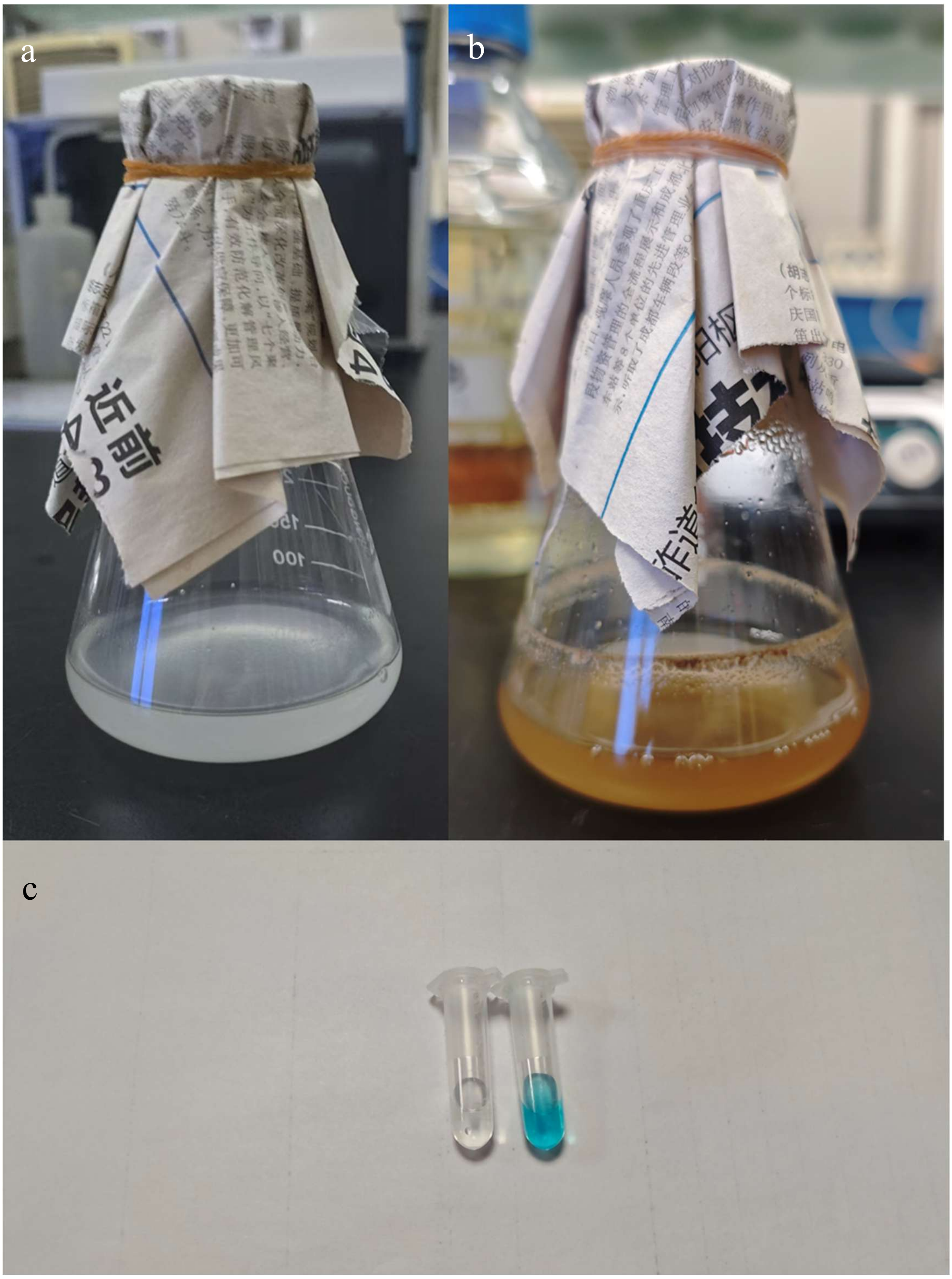

Figure S1. The initial (a) and 7th day (b) status of DT1 in PCYM medium; The BMO produced by DT1 caused LBB to change from transparent to blue (c).

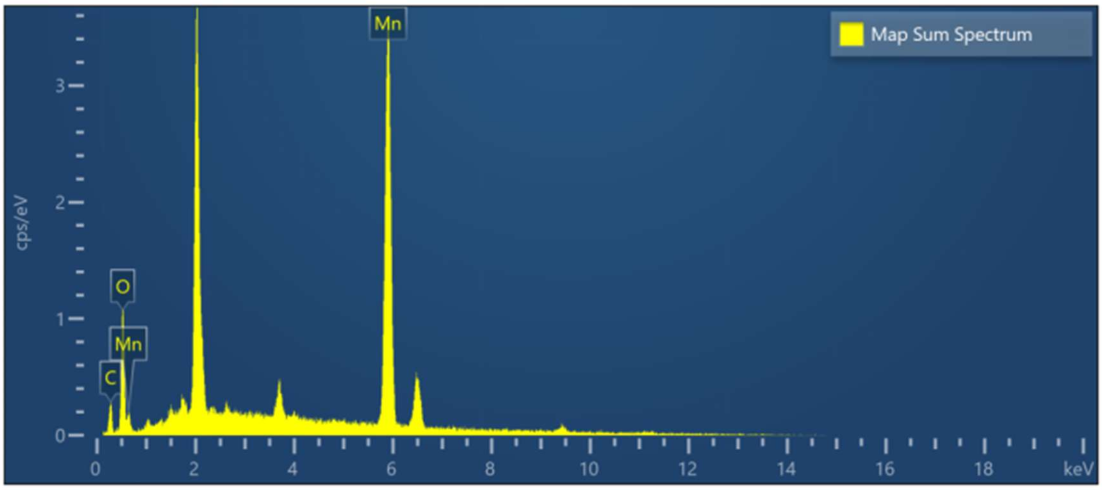

Figure S2. The EDS results of BMO.

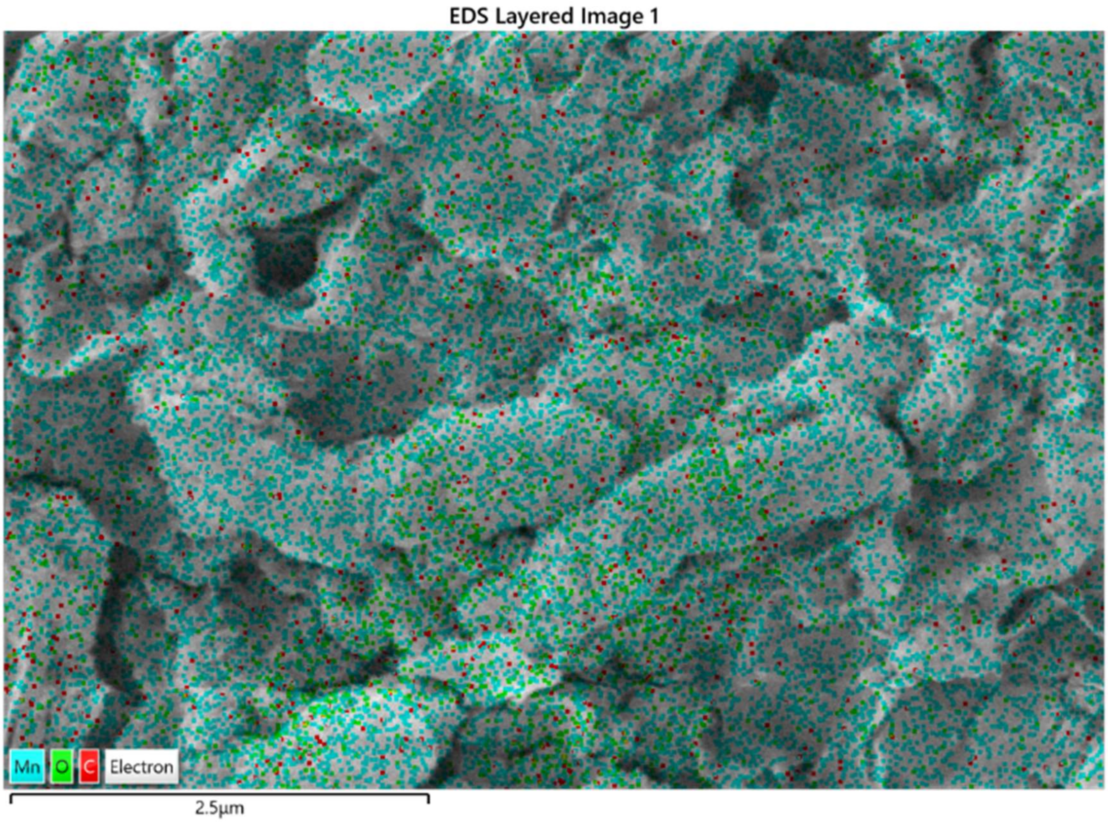

Figure S3. The mapping results of BMO.

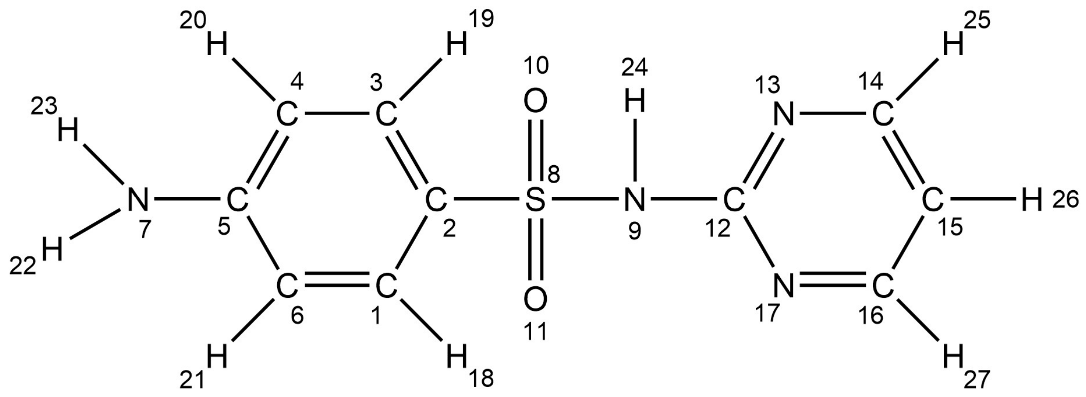

Figure S4. Atomic ordering of SDZ.

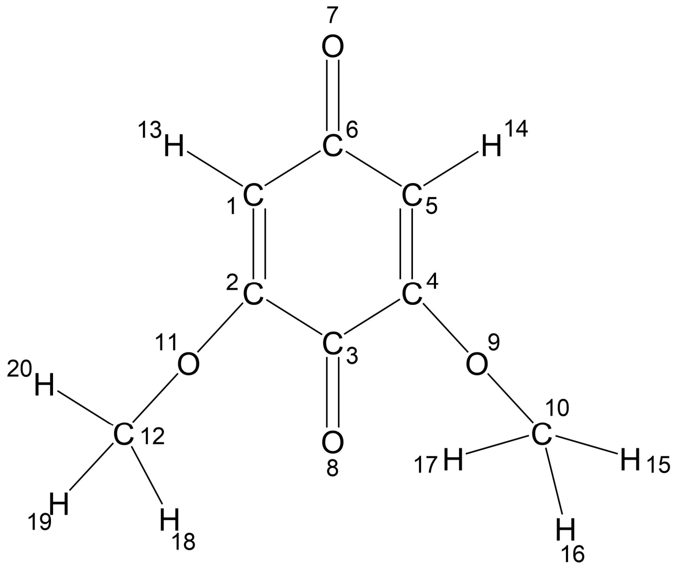

Figure S5. Atomic ordering of DMBQ.

a

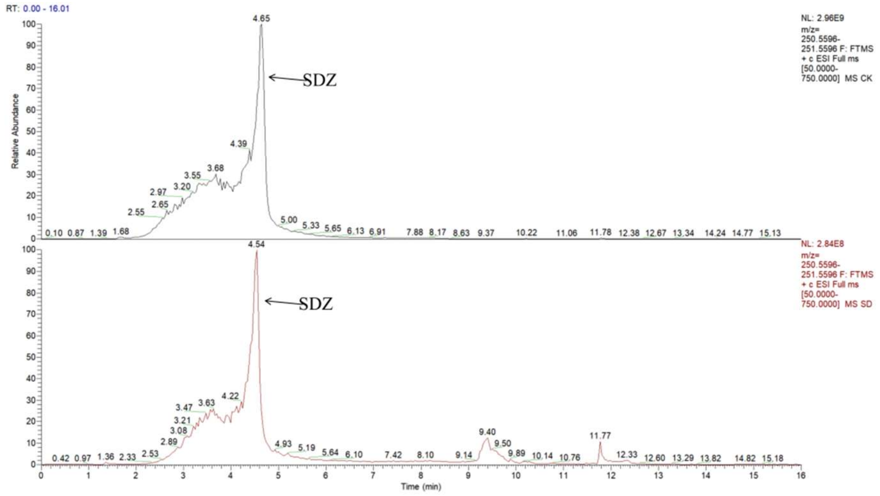

RT: 0.00 - 16.01
NL: 2.96E9
m/z= 250.5596-251.5596 F: FTMS + c ESI Full ms [50.0000-750.0000] MS CK
SDZ
NL: 2.84E8
m/z= 250.5596-251.5596 F: FTMS + c ESI Full ms [50.0000-750.0000] MS SD
SDZ
Relative Abundance
Time (min)

b

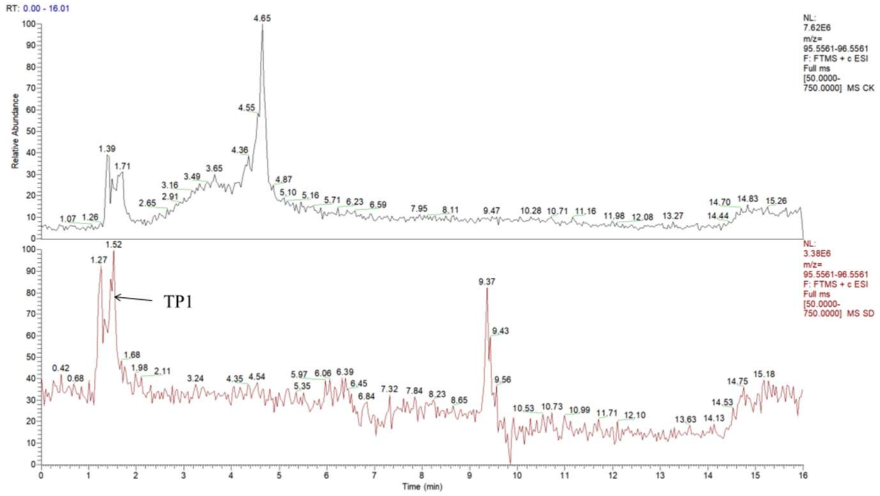

RT: 0.00 - 16.01
NL: 7.62E6
m/z= 95.5561-96.5561 F: FTMS + c ESI Full ms [50.0000-750.0000] MS CK
NL: 3.38E6
m/z= 95.5561-96.5561 F: FTMS + c ESI Full ms [50.0000-750.0000] MS SD
TP1
Relative Abundance
Time (min)

c

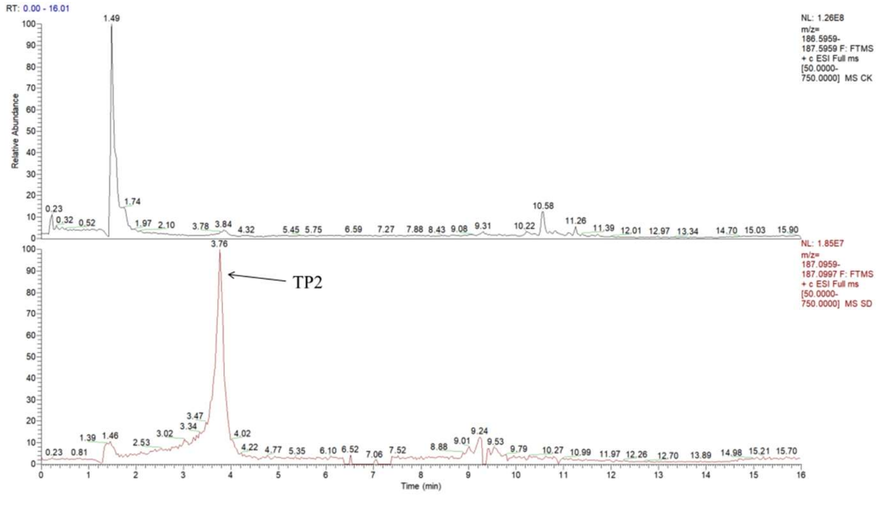

RT: 0.00 - 16.01
NL: 1.26E8
m/z= 186.5959-187.5959 F: FTMS + c ESI Full ms [50.0000-750.0000] MS CK
NL: 1.85E7
m/z= 187.0959-187.0997 F: FTMS + c ESI Full ms [50.0000-750.0000] MS SD
TP2
Relative Abundance
Time (min)

d

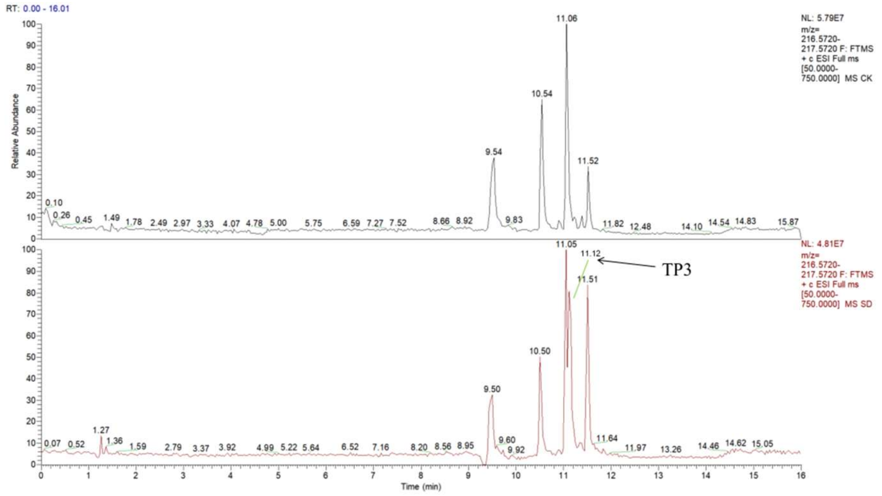
RT: 0.00 - 16.01
Relative Abundance
Time (min)
NL: 5.79E7
m/z= 216.5720-217.5720 F: FTMS + c ESI Full ms [50.0000-750.0000] MS CK
NL: 4.81E7
m/z= 216.5720-217.5720 F: FTMS + c ESI Full ms [50.0000-750.0000] MS SD
TP3

e

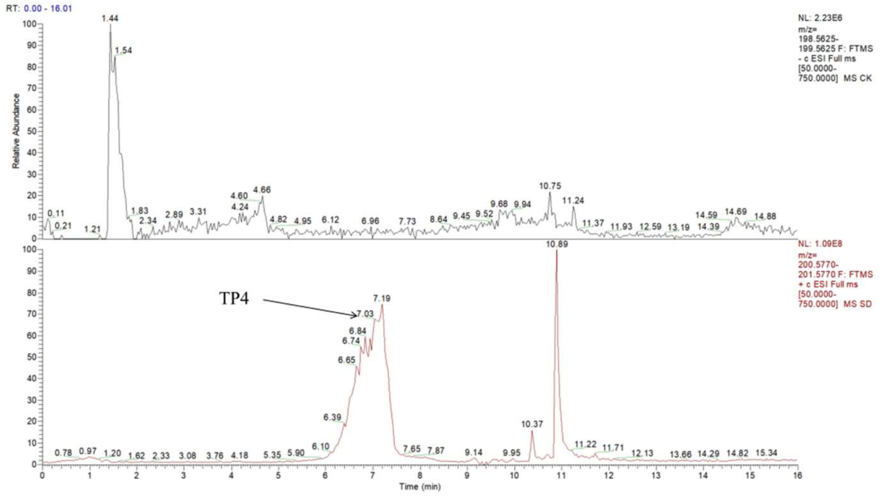
RT: 0.00 - 16.01
Relative Abundance
Time (min)
NL: 2.23E6
m/z= 198.5625-199.5625 F: FTMS - c ESI Full ms [50.0000-750.0000] MS CK
NL: 1.09E8
m/z= 200.5770-201.5770 F: FTMS + c ESI Full ms [50.0000-750.0000] MS SD
TP4

f

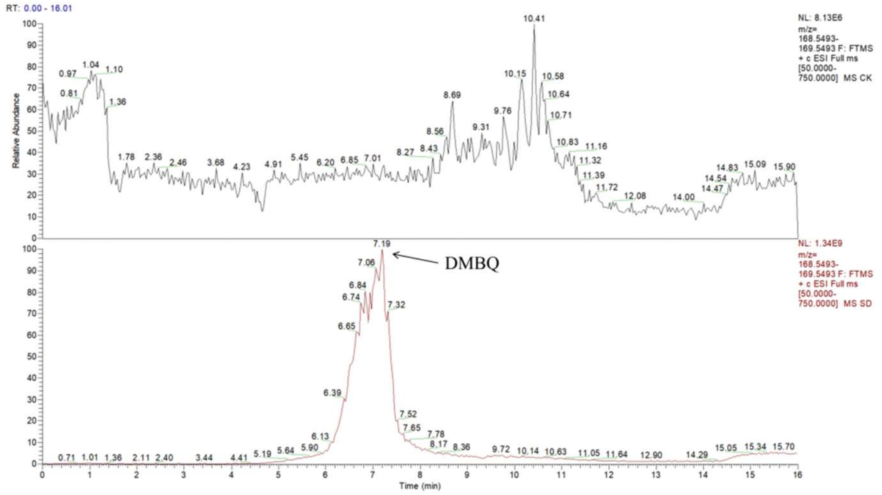
RT: 0.00 - 16.01
Relative Abundance
Time (min)
NL: 8.13E6
m/z= 168.5493-169.5493 F: FTMS + c ESI Full ms [50.0000-750.0000] MS CK
NL: 1.34E9
m/z= 168.5493-169.5493 F: FTMS + c ESI Full ms [50.0000-750.0000] MS SD
DMBQ

g

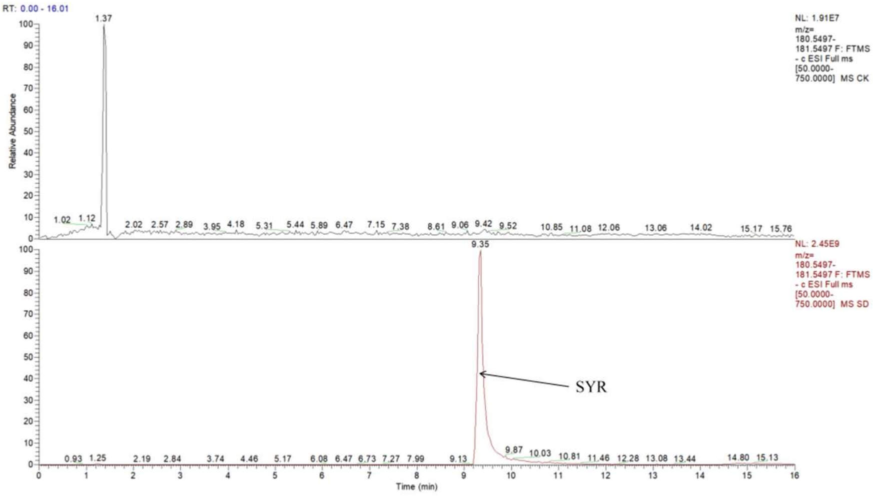

h

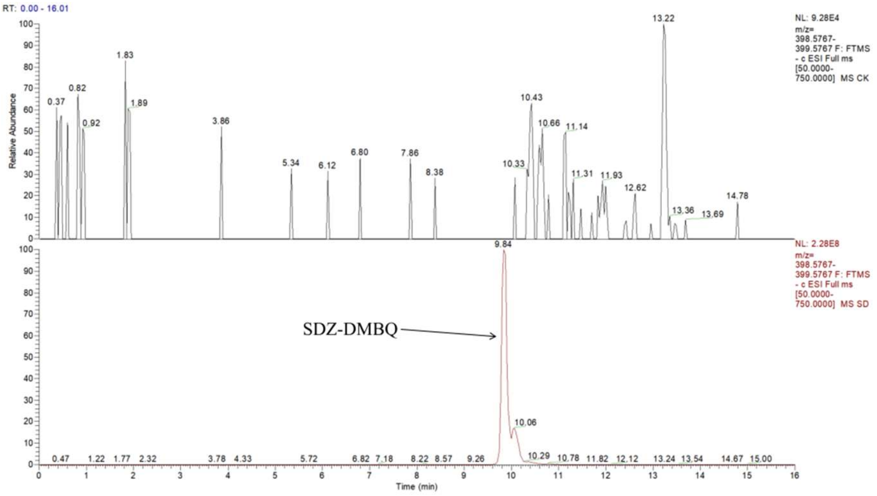

Figure S6. Total ion chromatograms of (a) SDZ, (b) TP1, (c) TP2, (d) TP3, (e) TP4, (f) DMBQ, (g) SYR, and (h) SDZ-DMBQ.

a

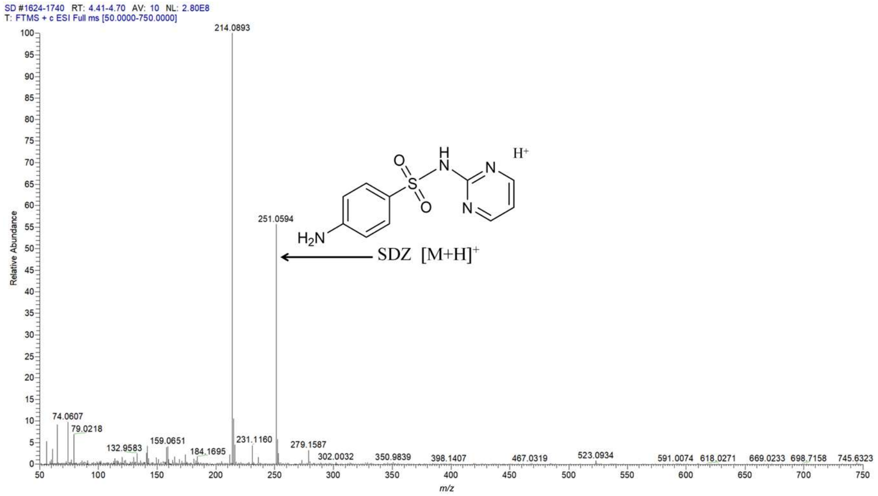
SD #1624-1740 RT: 4.41-4.70 AV: 10 NL: 2.80E8
T: FTMS + c ESI Full ms [50.0000-750.0000]
Relative Abundance
m/z
214.0893
251.0594
SDZ [M+H]+
H+
74.0607
79.0218
132.9583
159.0651
184.1695
231.1160
279.1587
302.0032
350.9839
398.1407
467.0319
523.0934
591.0074
618.0271
669.0233
698.7158
745.6323

b

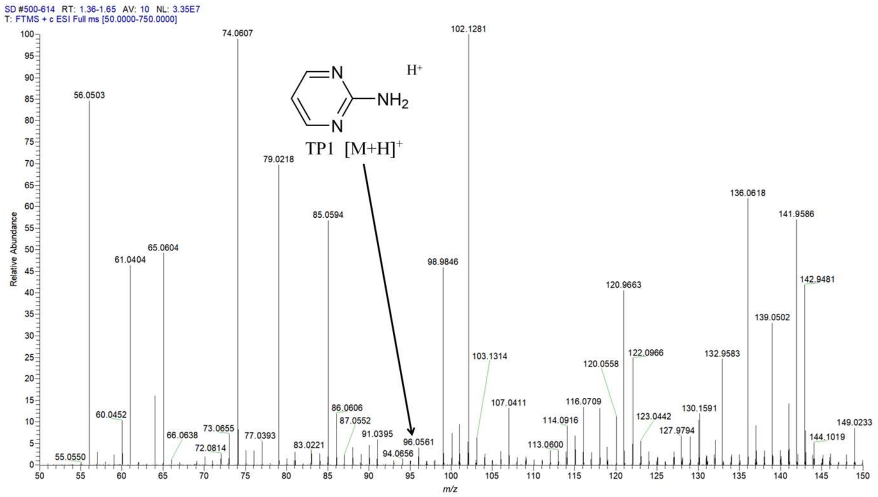
SD #500-614 RT: 1.36-1.65 AV: 10 NL: 3.35E7
T: FTMS + c ESI Full ms [50.0000-750.0000]
Relative Abundance
m/z
TP1 [M+H]+
H+
74.0607
102.1281
56.0503
79.0218
85.0594
136.0618
141.9586
65.0604
61.0404
98.9846
120.9663
142.9481
139.0502
103.1314
120.0558
122.0966
132.9583
107.0411
116.0709
114.0916
123.0442
130.1591
149.0233
60.0452
86.0606
87.0552
73.0655
66.0638
77.0393
91.0395
96.0561
83.0221
72.0814
94.0656
113.0600
127.9794
144.1019
55.0550

c

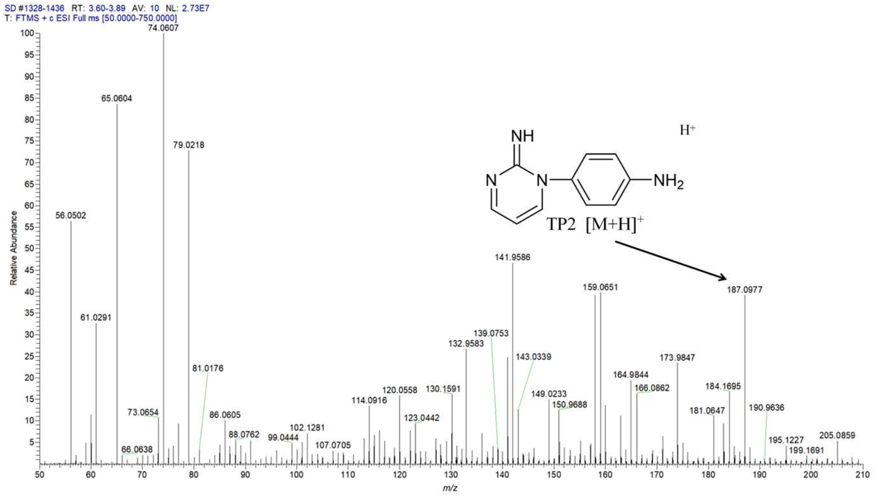
SD #1328-1436 RT: 3.60-3.89 AV: 10 NL: 2.73E7
T: FTMS + c ESI Full ms [50.0000-750.0000]
Relative Abundance
m/z
TP2 [M+H]+
H+
74.0607
65.0604
79.0218
56.0502
141.9586
159.0651
187.0977
61.0291
139.0753
132.9583
143.0339
173.9847
81.0176
164.9844
166.0862
184.1695
149.0233
150.9688
114.0916
120.0558
130.1591
181.0647
190.9636
73.0654
86.0605
123.0442
88.0762
102.1281
99.0444
107.0705
66.0638
195.1227
205.0859
199.1691

d

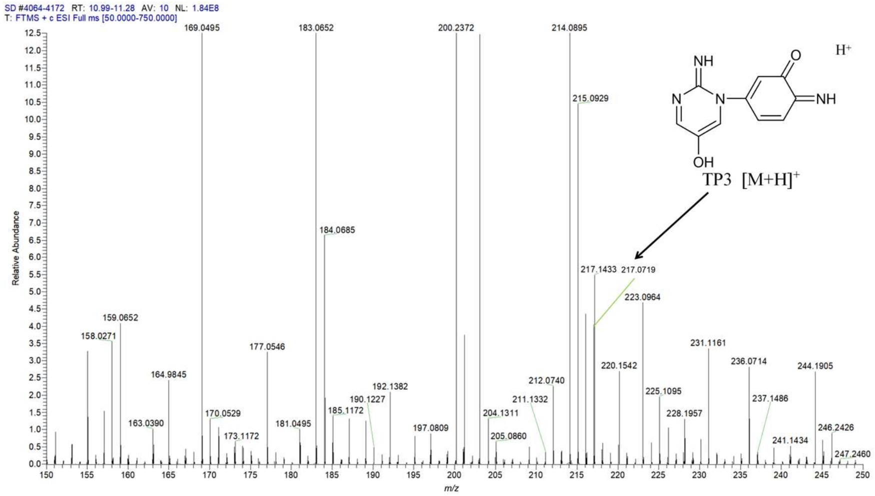

SD #4064-4172 RT: 10.99-11.28 AV: 10 NL: 1.84E8
T: FTMS + c ESI Full ms [50.0000-750.0000]
Relative Abundance
m/z
169.0495
183.0652
200.2372
214.0895
215.0929
184.0685
217.1433
217.0719
223.0964
159.0652
158.0271
177.0546
231.1161
236.0714
244.1905
220.1542
164.9845
212.0740
192.1382
225.1095
190.1227
237.1486
211.1332
170.0529
185.1172
204.1311
228.1957
163.0390
181.0495
197.0809
246.2426
173.1172
205.0860
241.1434
247.2460
H+
TP3 [M+H]+

e

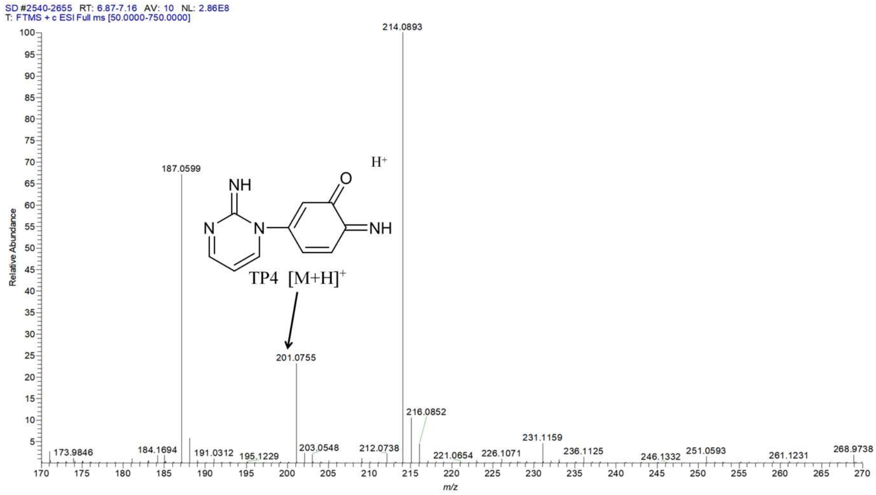

SD #2540-2655 RT: 6.87-7.16 AV: 10 NL: 2.86E8
T: FTMS + c ESI Full ms [50.0000-750.0000]
Relative Abundance
m/z
214.0893
187.0599
201.0755
216.0852
231.1159
173.9846
184.1694
191.0312
195.1229
203.0548
212.0738
221.0654
226.1071
236.1125
246.1332
251.0593
261.1231
268.9738
H+
TP4 [M+H]+

f

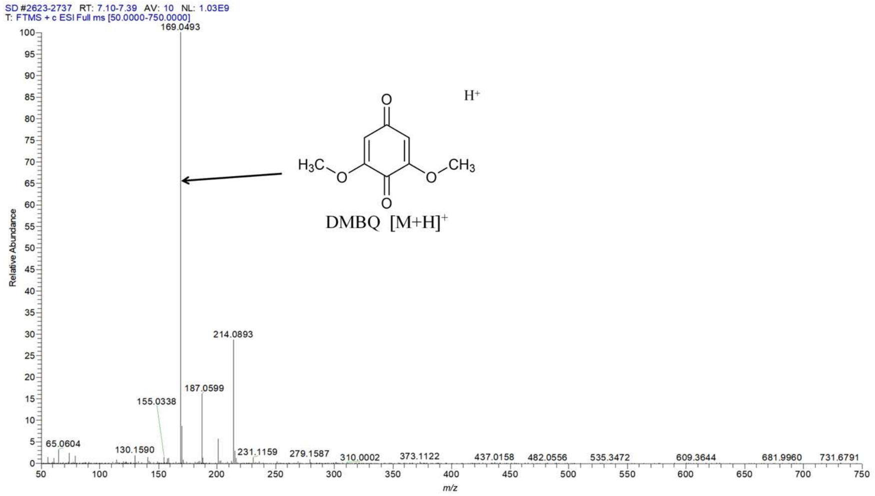

SD #2623-2737 RT: 7.10-7.39 AV: 10 NL: 1.03E9
T: FTMS + c ESI Full ms [50.0000-750.0000]
Relative Abundance
m/z
169.0493
214.0893
187.0599
155.0338
65.0604
130.1590
231.1159
279.1587
310.0002
373.1122
437.0158
482.0556
535.3472
609.3644
681.9960
731.6791
H+
DMBQ [M+H]+

g

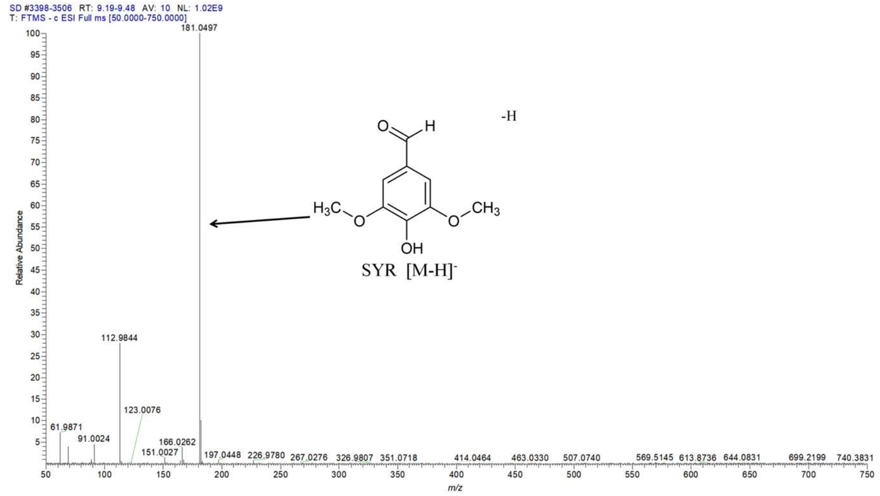

h

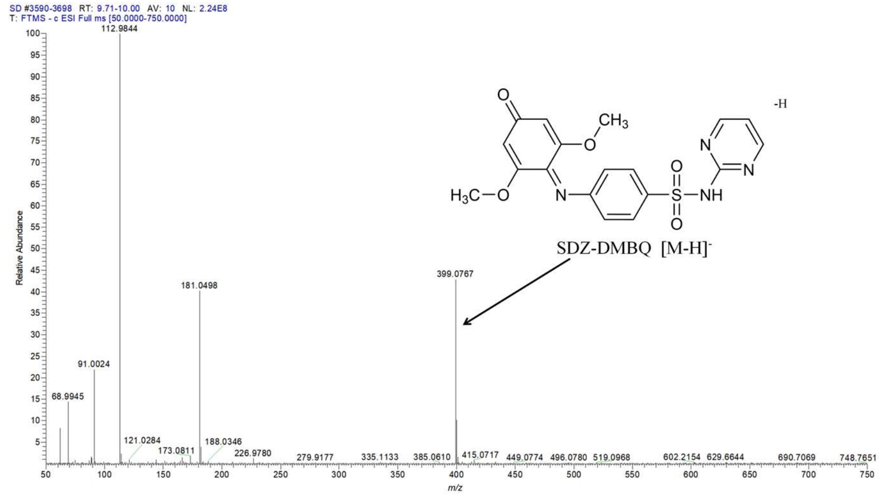

Figure S7. Mass spectrum of (a) SDZ, (b) TP1, (c) TP2, (d) TP3, (e) TP4, (f) DMBQ, (g) SYR, and (h) SDZ-DMBQ.

a

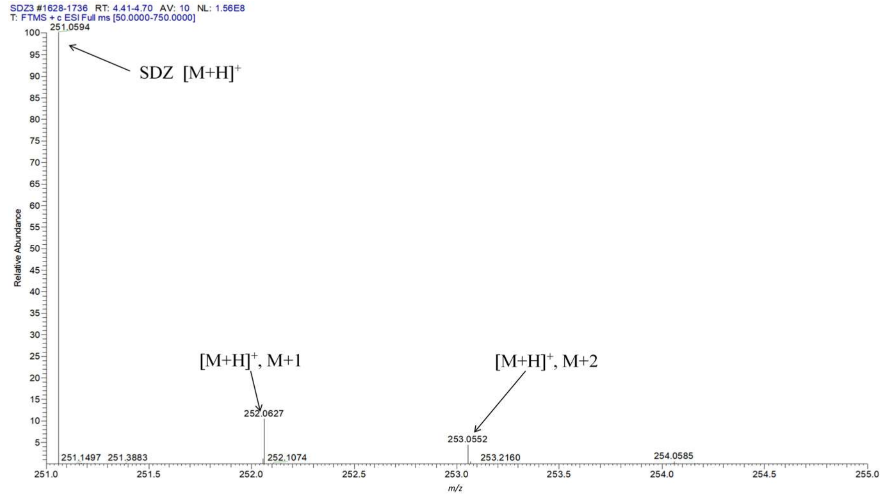

b

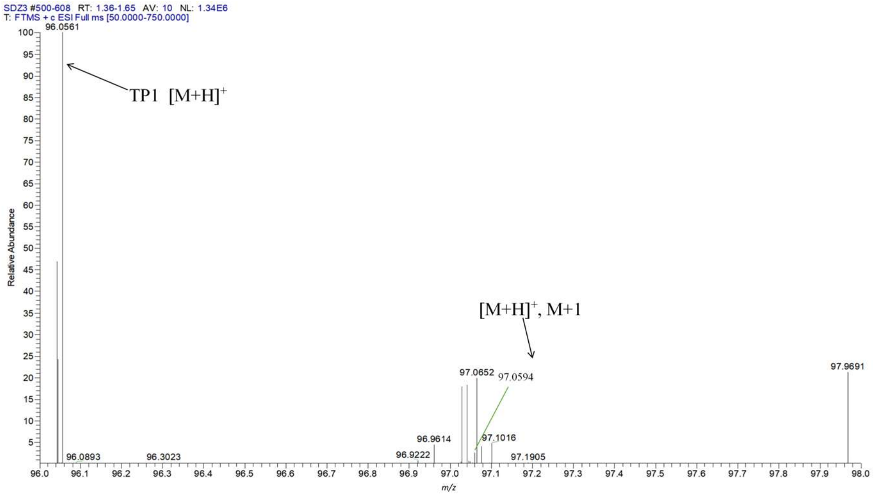
SDZ3 #500-608 RT: 1.36-1.65 AV: 10 NL: 1.34E6
T: FTMS + c ESI Full ms [50.0000-750.0000]
96.0561
TP1 [M+H]+
[M+H]+, M+1
97.0652
97.0594
97.9691
96.9614
97.1016
96.9222
96.0893
96.3023
97.1905
Relative Abundance
m/z
96.0 96.1 96.2 96.3 96.4 96.5 96.6 96.7 96.8 96.9 97.0 97.1 97.2 97.3 97.4 97.5 97.6 97.7 97.8 97.9 98.0

c

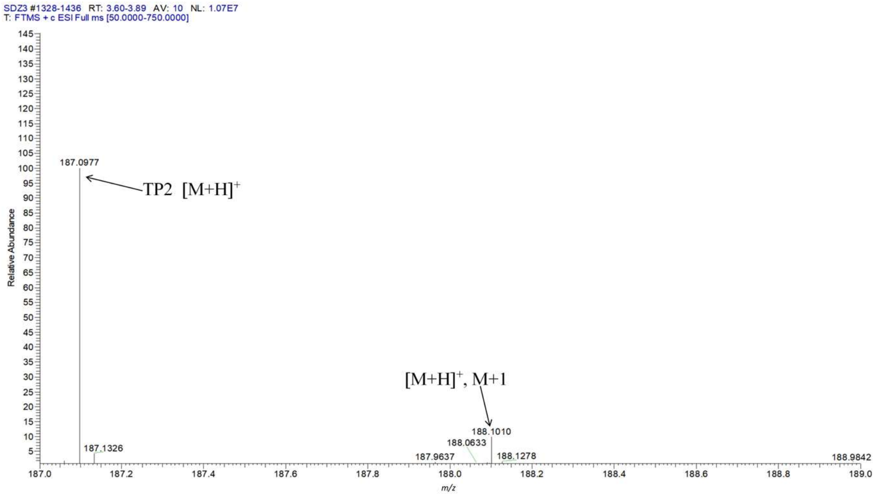
SDZ3 #1328-1436 RT: 3.60-3.89 AV: 10 NL: 1.07E7
T: FTMS + c ESI Full ms [50.0000-750.0000]
187.0977
TP2 [M+H]+
[M+H]+, M+1
188.1010
188.0633
187.1326
187.9637
188.1278
188.9842
Relative Abundance
m/z
187.0 187.2 187.4 187.6 187.8 188.0 188.2 188.4 188.6 188.8 189.0

d

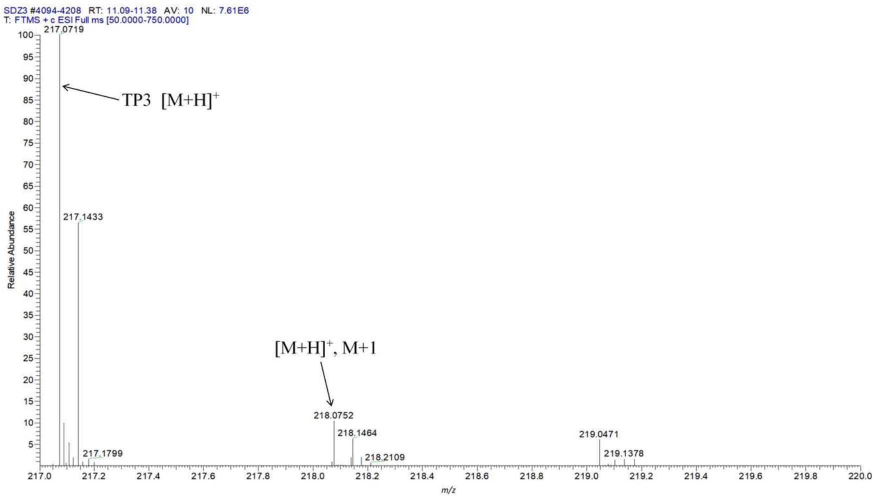
SDZ3 #4094-4208 RT: 11.09-11.38 AV: 10 NL: 7.61E6
T: FTMS + c ESI Full ms [50.0000-750.0000]
217.0719
TP3 [M+H]+
217.1433
[M+H]+, M+1
218.0752
218.1464
218.2109
217.1799
219.0471
219.1378
Relative Abundance
m/z
217.0 217.2 217.4 217.6 217.8 218.0 218.2 218.4 218.6 218.8 219.0 219.2 219.4 219.6 219.8 220.0

e

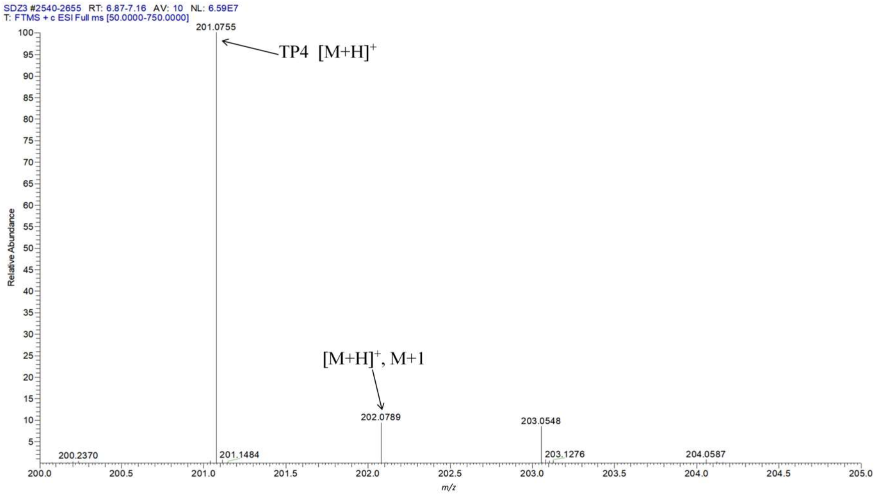
SDZ3 #2540-2655 RT: 6.87-7.16 AV: 10 NL: 6.59E7
T: FTMS + c ESI Full ms [50.0000-750.0000]
TP4 [M+H]+
[M+H]+, M+1
201.0755
202.0789
203.0548
200.2370
201.1484
203.1276
204.0587
Relative Abundance
m/z

f

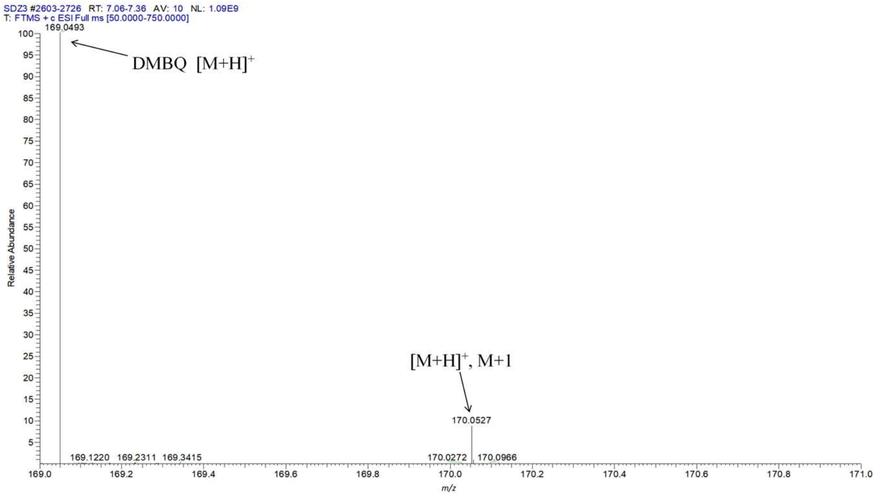
SDZ3 #2603-2726 RT: 7.06-7.36 AV: 10 NL: 1.09E9
T: FTMS + c ESI Full ms [50.0000-750.0000]
DMBQ [M+H]+
[M+H]+, M+1
169.0493
170.0527
169.1220
169.2311
169.3415
170.0272
170.0966
Relative Abundance
m/z

g

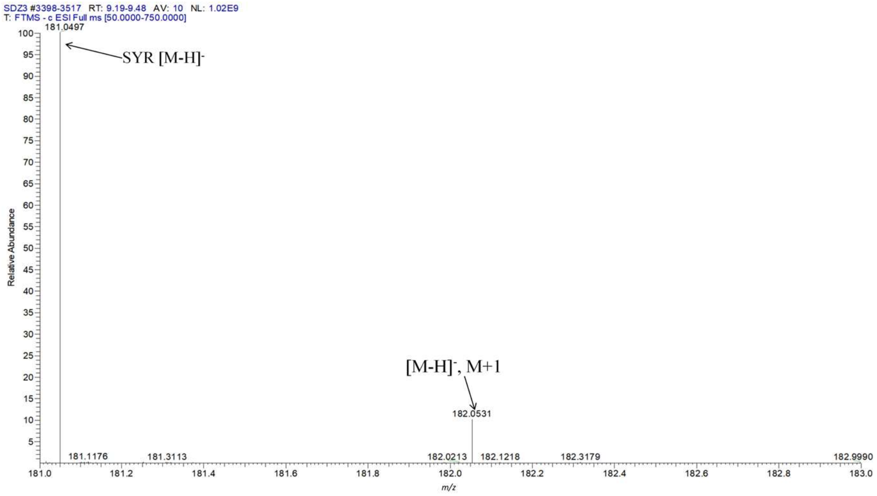
SDZ3 #3398-3517 RT: 9.19-9.48 AV: 10 NL: 1.02E9
T: FTMS - c ESI Full ms [50.0000-750.0000]
SYR [M-H]-
[M-H]-, M+1
181.0497
182.0531
181.1176
181.3113
182.0213
182.1218
182.3179
182.9990
Relative Abundance
m/z

h

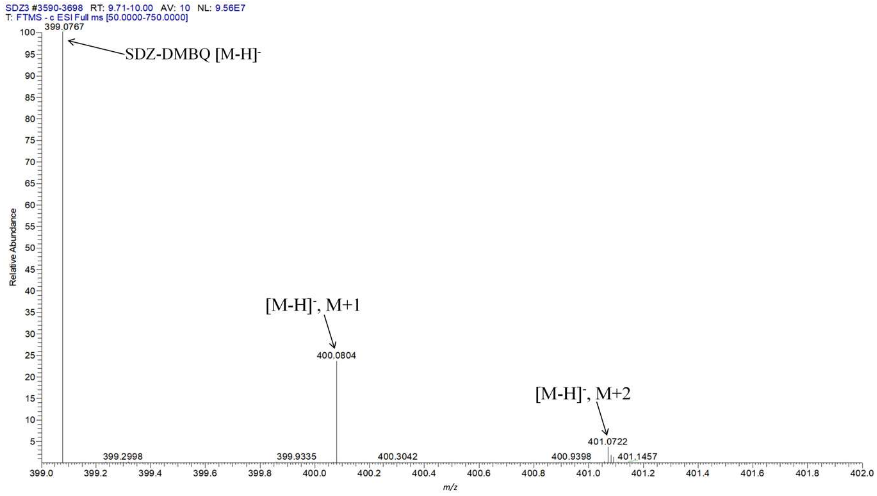

Figure S8. The observed isotopic patterns of (a) SDZ, (b) TP1, (c) TP2, (d) TP3, (e) TP4, (f) DMBQ, (g) SYR, and (h) SDZ-DMBQ.

a

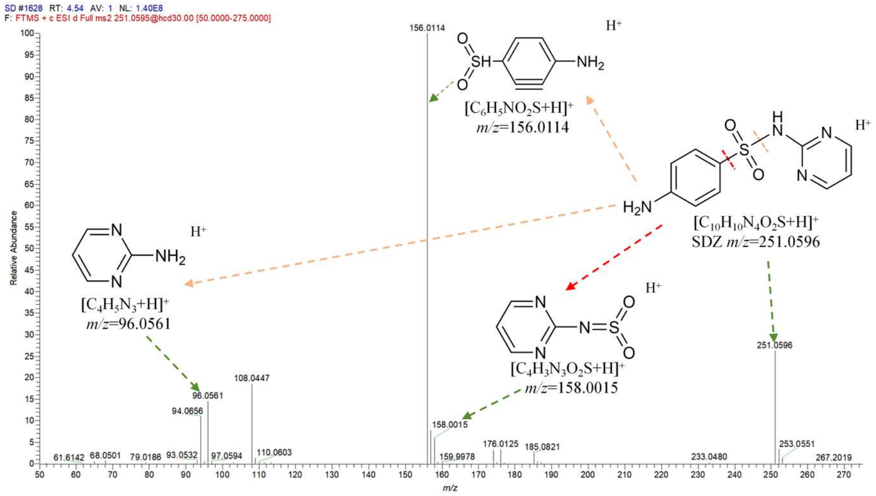

b

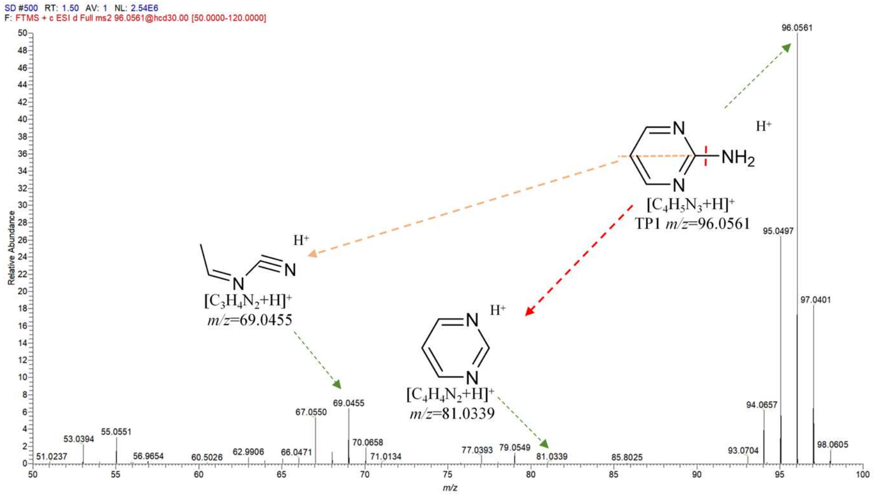

c

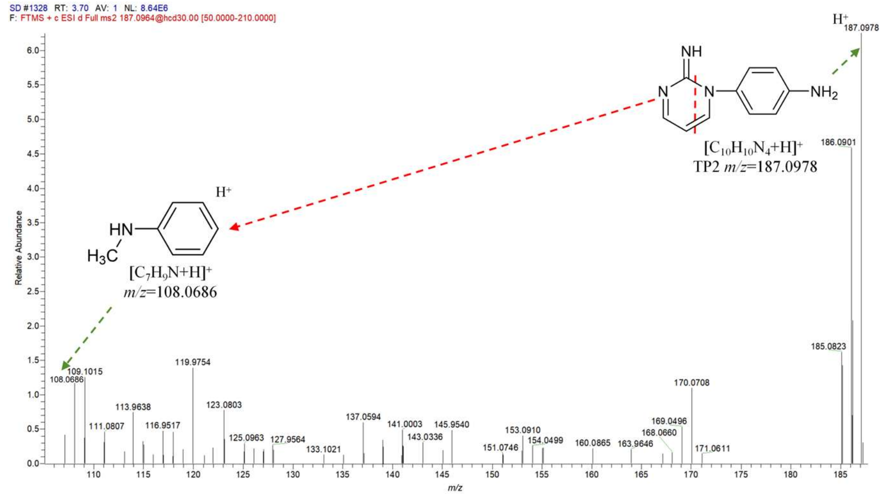
SD #1328 RT: 3.70 AV: 1 NL: 8.64E6
F: FTMS + c ESI d Full ms2 187.0964@hcd30.00 [50.0000-210.0000]
[C10H10N4+H]+
TP2 m/z=187.0978
[C7H9N+H]+
m/z=108.0686
Relative Abundance
m/z
187.0978
186.0901
185.0823
170.0708
169.0496
168.0660
171.0611
163.9646
160.0865
153.0910
154.0499
151.0746
145.9540
143.0336
141.0003
137.0594
133.1021
127.9564
125.0963
123.0803
119.9754
116.9517
113.9638
111.0807
109.1015
108.0686

d

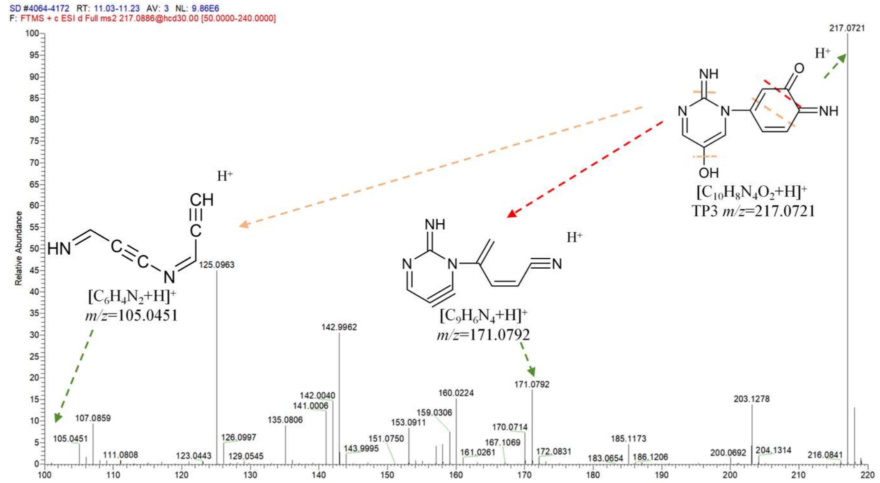
SD #4064-4172 RT: 11.03-11.23 AV: 3 NL: 9.86E6
F: FTMS + c ESI d Full ms2 217.0886@hcd30.00 [50.0000-240.0000]
[C10H8N4O2+H]+
TP3 m/z=217.0721
[C6H4N2+H]+
m/z=105.0451
[C9H6N4+H]+
m/z=171.0792
Relative Abundance
m/z
217.0721
216.0841
204.1314
203.1278
200.0692
186.1206
185.1173
183.0654
172.0831
171.0792
170.0714
167.1069
161.0261
160.0224
159.0306
153.0911
151.0750
143.9995
142.9962
142.0040
141.0006
135.0806
129.0545
126.0997
125.0963
123.0443
111.0808
107.0859
105.0451

e

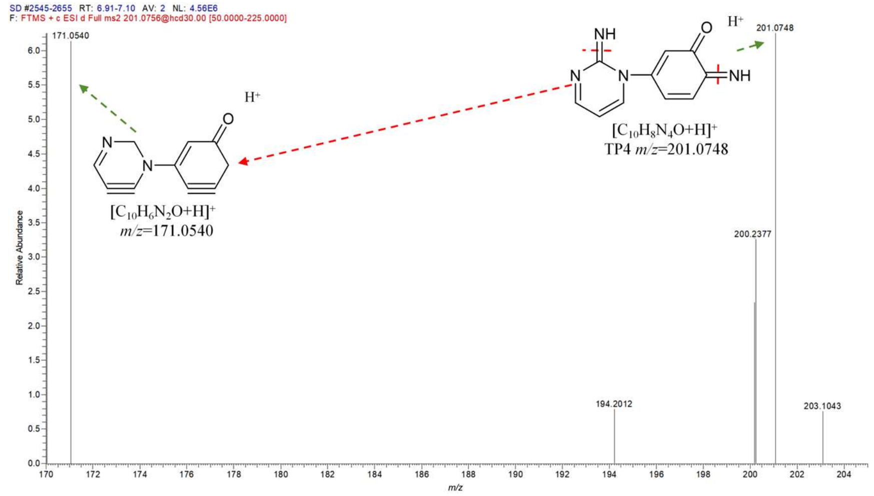
SD #2545-2655 RT: 6.91-7.10 AV: 2 NL: 4.56E6
F: FTMS + c ESI d Full ms2 201.0756@hcd30.00 [50.0000-225.0000]
[C10H8N4O+H]+
TP4 m/z=201.0748
[C10H6N2O+H]+
m/z=171.0540
Relative Abundance
m/z
171.0540
201.0748
200.2377
194.2012
203.1043

f

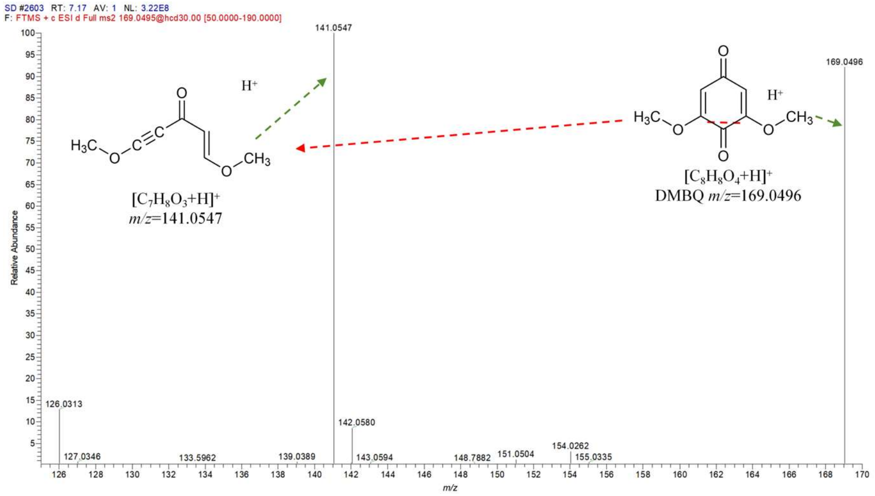

g

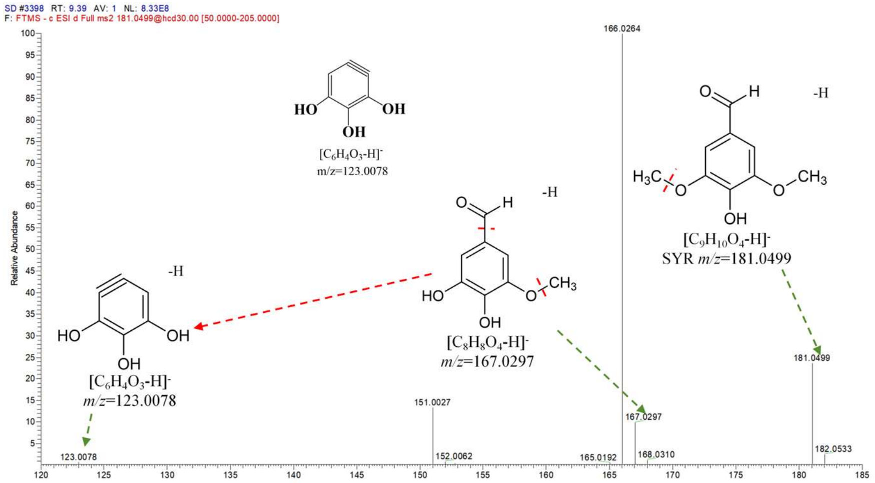

h

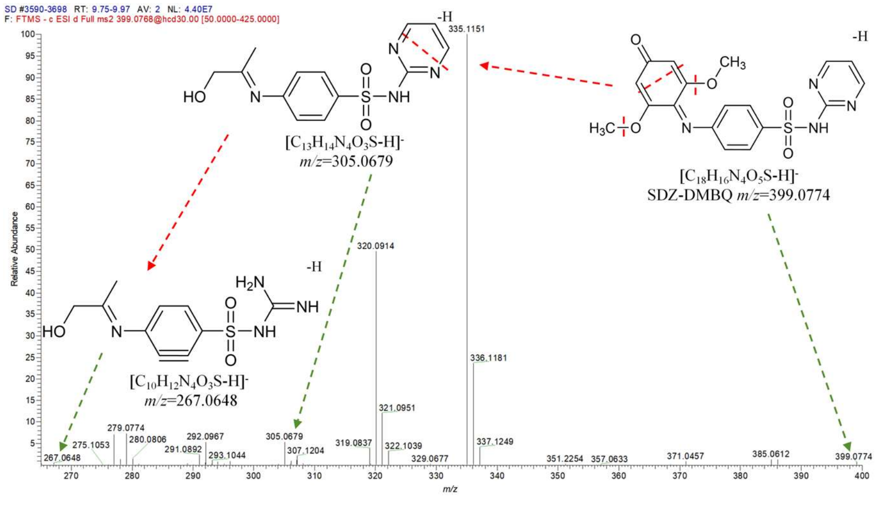

Figure S9. The MS/MS fragmentation profile and proposed fragmentation pattern of (a) SDZ, (b) TP1, (c) TP2, (d) TP3, (e) TP4, (f) DMBQ, (g) SYR, and (h) SDZ-DMBQ.

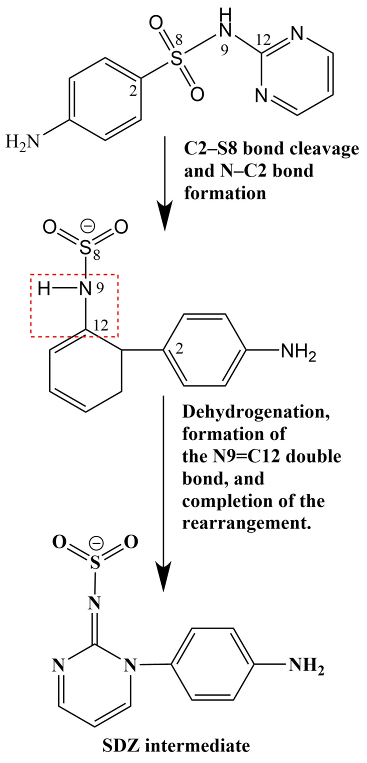

Figure S10. Schematic illustration of the Smiles rearrangement process of SDZ [12].

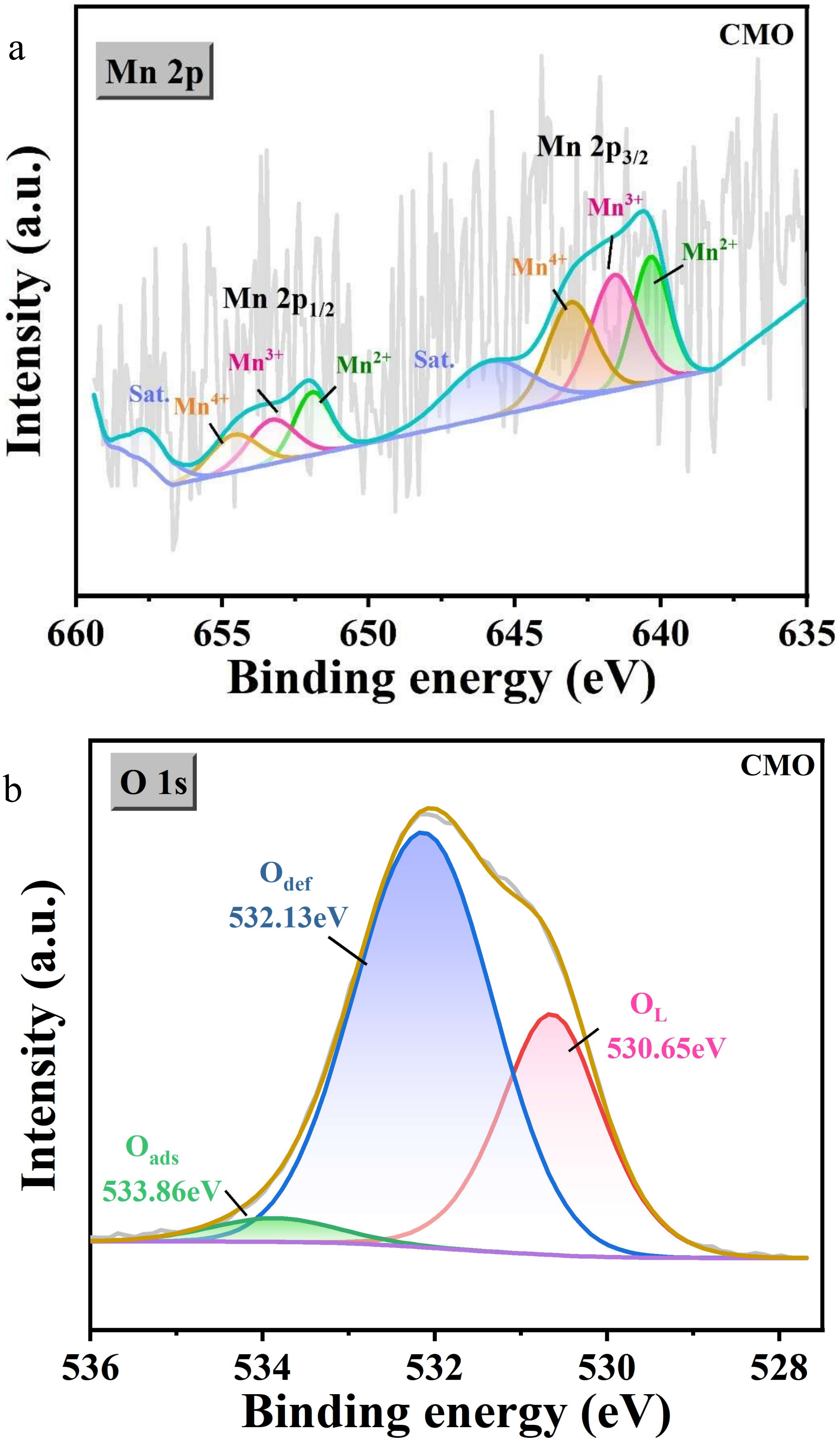
a
Mn 2p
CMO
Mn 2p$_{3/2}$
Mn^{3+}
Mn^{4+}
Mn^{2+}
Mn 2p$_{1/2}$
Mn^{3+}
Mn^{2+}
Sat.
Sat.
Mn^{4+}
Intensity (a.u.)
660
655
650
645
640
635
Binding energy (eV)
b
O 1s
CMO
O$_{def}$
532.13eV
O$_{L}$
530.65eV
O$_{ads}$
533.86eV
Intensity (a.u.)
536
534
532
530
528
Binding energy (eV)

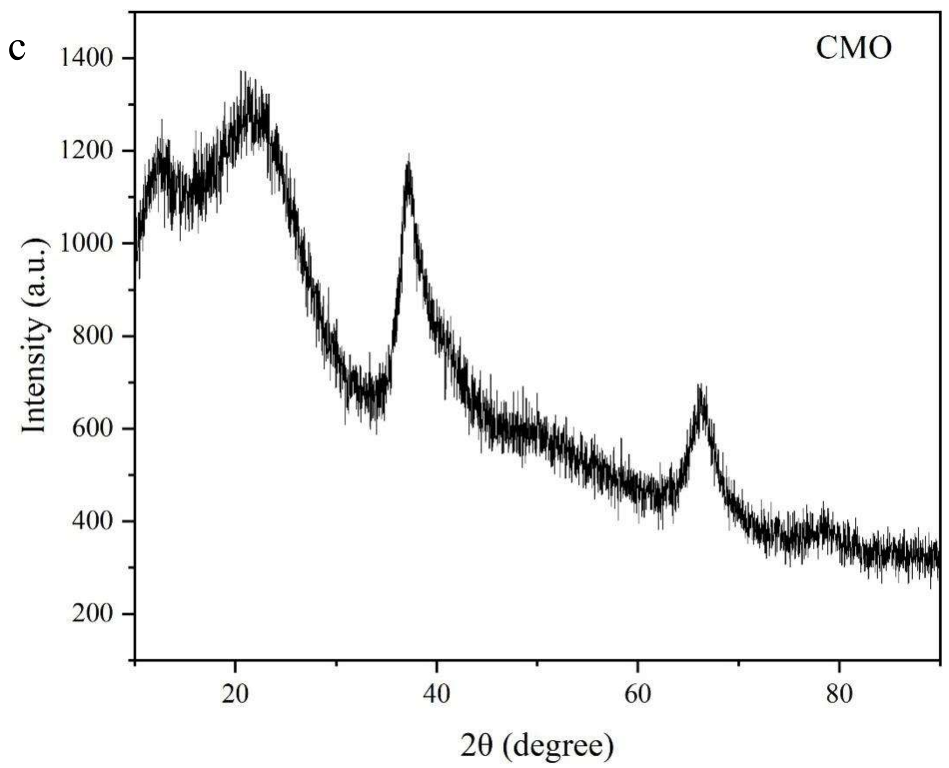

Figure S11. XPS spectra of Mn2p (a) and O1s (b) and XRD (c) of chemical synthesis of manganese oxide (CMO).

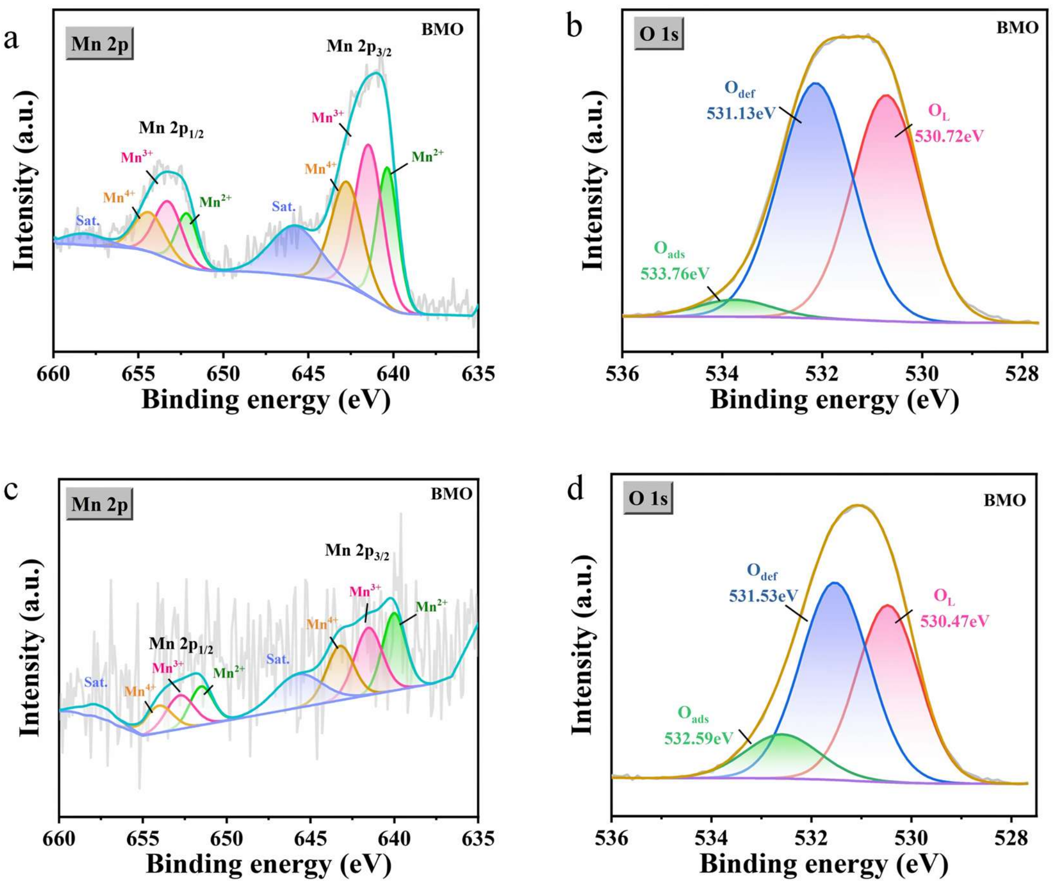

Figure S12. The difference of XPS of BMO before (a, b) and after cycling (c, d).

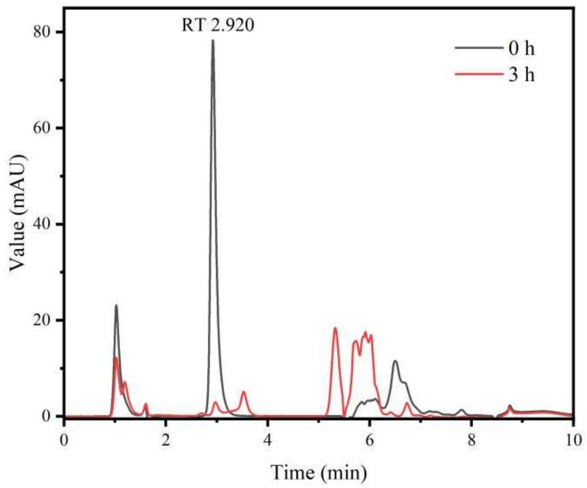

Figure S13. Representative HPLC chromatograms of SDZ before and after reaction in the BMO/SYR system.

References

1. Zhang, Q.; Tan, Y.; Chen, Y.; Xu, A.; Zhang, Y. Degradation of enrofloxacin with natural manganese oxides and enhancement by manganese oxidizing bacteria. Water Resources and Industry 2024, 31, 100257, doi:https://doi.org/10.1016/j.wri.2024.100257.
2. Liao, X.; Zhang, C.; Liu, Y.; Luo, Y.; Wu, S.; Yuan, S.; Zhu, Z. Abiotic degradation of methyl parathion by manganese dioxide: Kinetics and transformation pathway. Chemosphere 2016, 150, 90-96, doi:https://doi.org/10.1016/j.chemosphere.2016.02.028.
3. Das, R.; Liang, Z.; Li, G.; An, T. A non-blue laccase of Bacillus sp. GZB displays manganese-oxidase activity: A study of laccase characterization, Mn(II) oxidation and prediction of Mn(II) oxidation mechanism. Chemosphere 2020, 252, 126619, doi:https://doi.org/10.1016/j.chemosphere.2020.126619.
4. Liu, C.; Shi, B.; Guo, Y.; Wang, L.; Li, S.; Zhao, C.; Zhu, L.; Wang, J.; Kim, Y.M.; Wang, J. Characteristics of biological manganese oxides produced by manganese-oxidizing bacteria H38 and its removal mechanism of oxytetracycline. Environmental Pollution 2024, 345, 123432, doi:https://doi.org/10.1016/j.envpol.2024.123432.
5. Song, Y.; Jiang, J.; Ma, J.; Zhou, Y.; von Gunten, U. Enhanced transformation of sulfonamide antibiotics by manganese(IV) oxide in the presence of model humic constituents. Water Research 2019, 153, 200-207, doi:https://doi.org/10.1016/j.watres.2019.01.011.
6. Guo, W.; Yang, Z.; Du, J.; Yin, R.; Zhou, X.; Jin, S.; Ren, N. Degradation of sulfadiazine in water by a UV/O3 process: performance and degradation pathway. RSC Advances 2016, 6, 57138-57143, doi:10.1039/C6RA09078H.
7. Zhu, L.; Shi, Z.; Deng, L.; Duan, Y. Efficient degradation of sulfadiazine using magnetically recoverable MnFe2O4/δ-MnO2 hybrid as a heterogeneous catalyst of peroxymonosulfate. Colloids and Surfaces A: Physicochemical and Engineering Aspects 2021, 609, 125637, doi:https://doi.org/10.1016/j.colsurfa.2020.125637.
8. Song, Y.; Yu, Y.; Jin, M.; Hou, C.; Wang, J.; Wang, X.; Zhou, X.; Chen, J.; Shen, Z.; Zhang, Y. Sulfadiazine removal efficiency with persulfate driven by electron-rich Cu-beta zeolites. Chemosphere 2023, 344, 140300, doi:https://doi.org/10.1016/j.chemosphere.2023.140300.
9. Wang, Y.; Gan, T.; Xiu, J.; Liu, G.; Zou, H. Degradation of sulfadiazine in aqueous media by peroxymonosulfate activated with biochar-supported ZnFe2O4 in combination with visible light in an internal loop-lift reactor. RSC Advances 2022, 12, 24088-24100, doi:10.1039/D2RA04573G.
10. Leng, Y.; Xiao, H.; Li, Z.; Liu, Y.; Wang, J. Transformation of sulfadiazine in humic acid and polystyrene microplastics solution by horseradish peroxidase coupled with 1-hydroxybenzotriazole. Chemosphere 2021, 269, 128705, doi:https://doi.org/10.1016/j.chemosphere.2020.128705.
11. Leng, Y.; Zhang, Y.; Chen, X.; He, T.; Chang, F.; Li, Z.; Huang, Y.; Wang, J.; Xiong, W. Enhanced transformation of sulfadiazine by horseradish peroxidase activated persulfate progress: Characteristics, pathways, mechanisms, and ecotoxicities. Journal of Environmental Chemical Engineering 2025, 13, 116140, doi:https://doi.org/10.1016/j.jece.2025.116140.
12. Yang, L.; Shi, Y.; Li, J.; Fang, L.; Luan, T. Transformation of aqueous sulfonamides under horseradish peroxidase and characterization of sulfur dioxide extrusion products from sulfadiazine. Chemosphere 2018, 200, 164-172, doi:https://doi.org/10.1016/j.chemosphere.2018.01.118.
